# Rare but specific: 5-bp composite motifs define SMAD binding in BMP signaling

**DOI:** 10.1186/s12915-025-02183-1

**Published:** 2025-03-13

**Authors:** Jerome Jatzlau, Sophie-Nhi Do, Rebeca A. Mees, Paul-Lennard Mendez, Rameez Jabeer Khan, Lukas Maas, Lidia Ruiz, Pau Martin-Malpartida, Maria J. Macias, Petra Knaus

**Affiliations:** 1 Institute of Chemistry and Biochemistry, Freie Universitaet Berlin, Berlin, Germany; 2 Institute for Research in Biomedicine (IRB Barcelona), The Barcelona Institute of Science and Technology (BIST), Baldiri Reixac, 10, Barcelona, 08028 Spain; 3 ICREA, Passeig Lluís Companys 23, Barcelona, 08010 Spain

**Keywords:** BMP, Signal transduction, SMAD1/5/8, Target gene regulation, Composite motifs

## Abstract

**Background:**

Receptor-activated SMADs trimerize with SMAD4 to regulate context-dependent target gene expression. However, the presence of a single SMAD1/5/8 binding motif in cis-regulatory elements alone does not trigger transcription in native contexts. We hypothesize that binding to composite motifs in which at least two SMAD binding sites are in close proximity would be enough to induce transcription as this scenario allows the simultaneous interaction of at least two SMAD proteins, thereby increasing specificity and affinity.

**Results:**

Using more than 65 distinct firefly luciferase constructs, we delineated the minimal requirements for BMP-induced gene activation. We propose a model in which two SMAD-MH1 domains bind a SMAD-composite motif in a back-to-back fashion with a 5-bp distance between the SMAD-motifs on opposing DNA strands. However screening of SMAD1-bound regions across a variety of cell types highlights that these composite motifs are extremely uncommon, explaining below 1% of SMAD1 binding events.

**Conclusions:**

Deviations from these minimal requirements prevent transcription and underline the need for co-transcription factors to achieve gene activation.

**Supplementary Information:**

The online version contains supplementary material available at 10.1186/s12915-025-02183-1.

## Background

Balanced signaling of bone/body morphogenetic protein (BMP) and transforming growth factor β (TGFβ) is crucial for tissue differentiation and maintenance [1], while dysregulated signaling correlates with the initiation and progression of various diseases [2–5]. SMAD transcription factors (TFs) act as key mediators downstream of the BMP and TGFβ family of cytokines [6]. SMADs are cytosolic proteins carrying an N-terminal DNA-binding domain (MH1) connected via a flexible linker region to a C-terminal domain (MH2). The MH2 domain serves as an interaction platform for BMP/TGFβ receptors, other SMADs, or co-transcription factors (coTFs) [6, 7]. BMP/TGFβ ligand binding to hetero-tetrameric transmembrane serine/threonine kinase receptor complexes triggers SMAD phosphorylation at the C-terminus, thereby activating specific receptor-SMADs (R-SMADs) [8]. Whereas BMP-stimulation leads to phosphorylation of SMAD1/5/8, TGFβ ligands signal via activation of SMAD2/3 [6]. Phosphorylation also facilitates MH2 dimerization/trimerization of R-SMADs with co-SMAD4 [9], leading to nuclear translocation. Once SMAD complexes are in the nucleus, binding to cis-regulatory elements (CREs) regulates target gene transcription [10]. The diverse biological outcomes of BMP and TGF-β signaling are orchestrated by coTF-mediated recruitment of SMADs to specific CREs in a cell type- and context-dependent manner [11–14].

Crystallization experiments revealed a shared DNA binding mode for all SMAD MH1 domains. Known SMAD recognition motifs include the first described palindromic GTCT*AGAC SMAD binding element (pSBE) and a palindromic GC-rich SBE (GGC*GCC; pGC-SBE). Other non-palindromic GC-rich SBEs include GGCTCC (npGC-SBE) [13, 15–18] and the 5GC motifs GGC(GC)|(CG) [19]. These binding motifs are enriched in BMP and TGFβ-sensitive SMAD-bound regions (SBRs) as identified by ChIP-seq [13, 16, 19]. The interaction of MH1 domains with the pSBE and with 5GC motifs has been described in several distinct crystallization studies [15, 19–22]. Binding to DNA occurs through contacts between a conserved β-hairpin in the MH1 domain and the major groove of the DNA. The β-hairpin contains a conserved Arg that interacts with the Gua in + 1 position of an SBE (GTCT) on the sense strand and a Lys that interacts with the Gua complementary to the Cyt in + 3 position [15, 19–22].

Despite the capacity of SMAD proteins to recognize and bind specific DNA motifs independently [19–24], the occurrence of single motifs cannot fully explain the distinct downstream responses to BMP and TGFβ inputs. One hypothesis is that, given their adoption of quaternary structures, SMADs can bind CREs as dimers or trimers, enabling simultaneous recognition of multiple DNA binding sites. Recent findings indicate that the precise orientation, spacing, and number of TF binding sites influence transcriptional responsiveness and enhancer function [25], confirming the “enhanceosome model” [26, 27]. This model can also describe transcriptional regulation by the SMAD TFs. For SMAD1/5/8, DNA binding occurs either through the recognition of GC-rich motifs (p/npGC-SBE or 5GC motif) [13, 19] or heterocomposite motifs [16], with the distance between these motifs influencing responsiveness to activated SMAD1/5/8 proteins.

Whereas the limitations of TGFβ-sensitive SMAD2/3 DNA binding and transcriptional regulation were studied previously [28], an comparative unbiased approach to investigate the minimal sequence requirements of cis-regulatory elements for BMP-dependent SMAD1/5/8 signaling was missing. Here we studied the correlation between the presence of the SMAD1/5/8 protein complex, DNA motif separation, and transcriptional activation in the absence of DNA sites for co-transcriptional activators. Through a comprehensive approach involving DNA binding studies, structural modeling, reporter gene assays, and motif enrichment studies, we elucidate the minimal requirements for BMP-sensitive SMAD1/5/8 transcription, revealing the need for binding of two SMADs to adjacent DNA motifs on opposing DNA strands in a back-to-back configuration.

## Results

### MH1 domain binding affinities to different DNA motifs

**Fig. 1:**
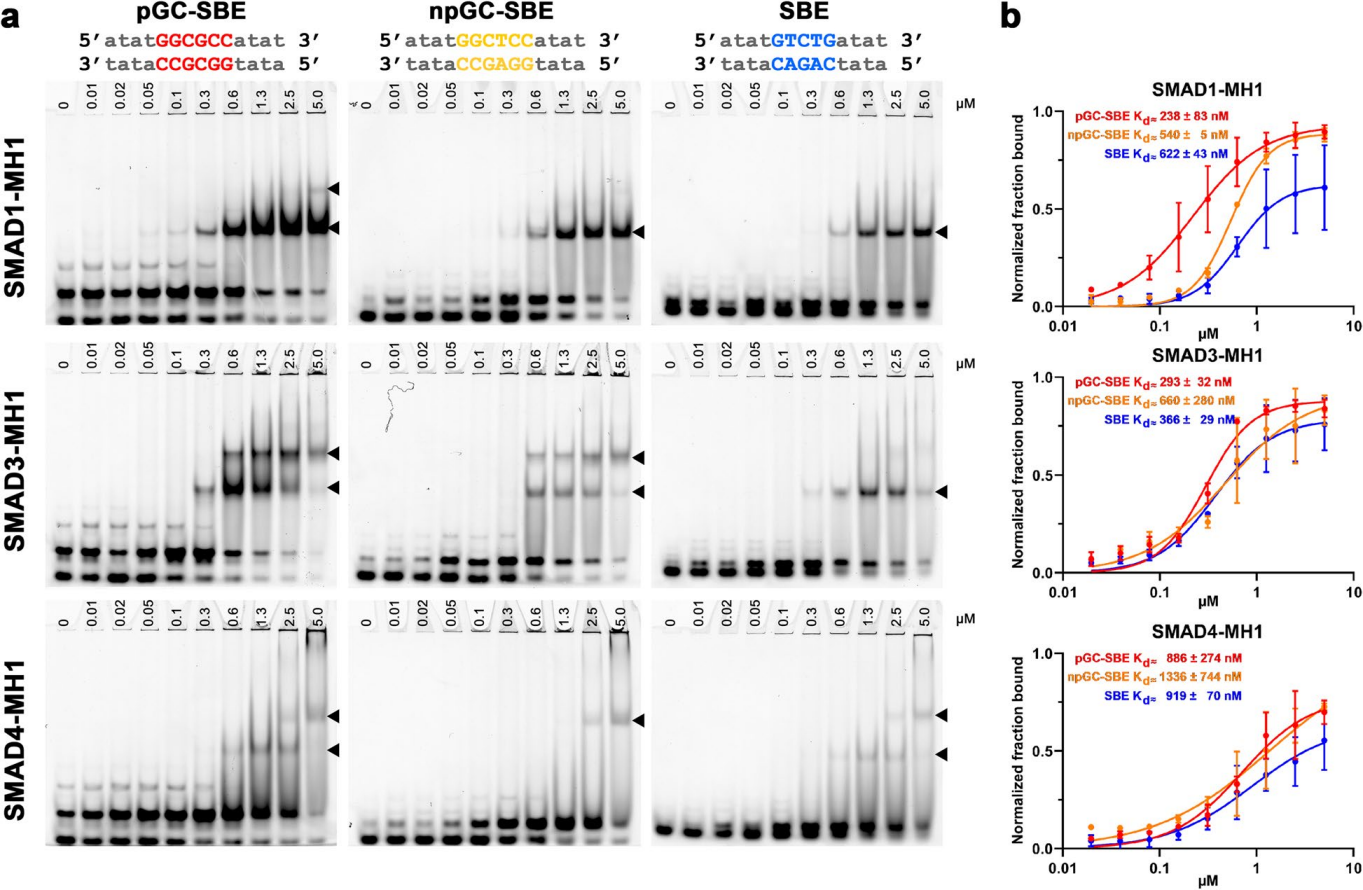
SMAD-MH1 domains bind different SMAD binding elements with similar affinities: **a** EMSA experiments are performed testing the binding of human SMAD1/3/4-MH1 domains to palindromic GC-SBE (pGC-SBE), non-palindromic GC-SBE (npGC-SBE), and SBE. Protein concentrations (µM) are shown on top of the EMSA. Abbreviations for the DNA oligonucleotides and dsDNA sequence are shown above. Single and double SMAD-MH1 binding to dsDNA is indicated with black triangles. Double binding at saturating concentrations (above 1.3 µM) can be the result of unspecific binding (representative blot). **b** Fractions of bound SBE probes plotted against SMAD-MH1 concentrations, with Hill equation curve fitting of data obtained. *K*_d_ values are indicated; *n* = 2 independent experiments

To assess the binding affinities of SMAD1/3/4 MH1 domains to the pGC-SBE (GGCGCC), npGC-SBE (GGCTCC), and SBE (GTCTG) motifs, we constructed three 14/15-bp Cy5-labeled dsDNA oligonucleotides, each containing one of the specified motifs, and we conducted electrophoretic mobility shift assays (EMSAs) (Additional file 1: Fig. S1). Our results demonstrated that all MH1 domains bound to each motif, but with varying affinities (Fig. 1a). Further, the bound fraction of MH1 domains was quantified, and Hill equation fitting was used to estimate binding affinities (Fig. 1b). We found the affinities of all tested SMAD MH1 domains for each of these motif to be in the upper nanomolar range, reflecting the results of previous SMAD-MH1 affinities determined by isothermal titration calorimetry (ITC) measurements [19]. SMAD1 MH1 showed relatively stronger binding to pGC-SBE^+^ (*K*_d_ = 238 ± 83 nM) compared to npGC-SBE^+^ (*K*_d_ = 540 ± 5 nM) and SBE^+^ (*K*_d_ = 622 ± 43 nM) oligonucleotides. Conversely, SMAD3 MH1 displayed the highest affinity for pGC-SBE^+^ (*K*_d_ = 293 ± 32 nM) and SBE^+^ oligonucleotides (*K*_d_ = 366 ± 29 nM) and a lower affinity for npGC-SBE^+^ (*K*_d_ = 660 ± 280 nM) ones. SMAD4 MH1 displayed the weakest affinity of the three tested MH1 domains with estimated affinities in the high nanomolar range (pGC-SBE^+^, *K*_d_ = 886 ± 274 nM; npGC-SBE^+^, *K*_d_ = 1336 ± 744 nM; SBE^+^, *K*_d_ = 919 ± 70 nM). Further, clear double binding to pGC-SBE was only observed for SMAD3-MH1 starting at low protein concentrations (0.6 µM). This is in line with previous reports where SMAD3-MH1 exhibited double binding to palindromic binding elements [20]. Hill equation fitting suggested a moderate positive cooperative binding to pGC-SBE^+^ oligonucleotides for SMAD3-MH1 (*h* = 1.80 ± 0.23) and only weak positive cooperative binding for SMAD1-MH1 and SMAD4-MH1 (*h* < 1.3).

Overall, while all MH1 domains showed moderate nanomolar binding affinities, BMP-sensitive SMAD1-MH1 possessed the highest binding affinity to pGC-SBE, in line with enrichment of this motif in SMAD1-bound regions identified by ChIP-Seq [13, 16].

### Defining the minimal SMAD1/5 responsive composite motif

**Fig. 2:**
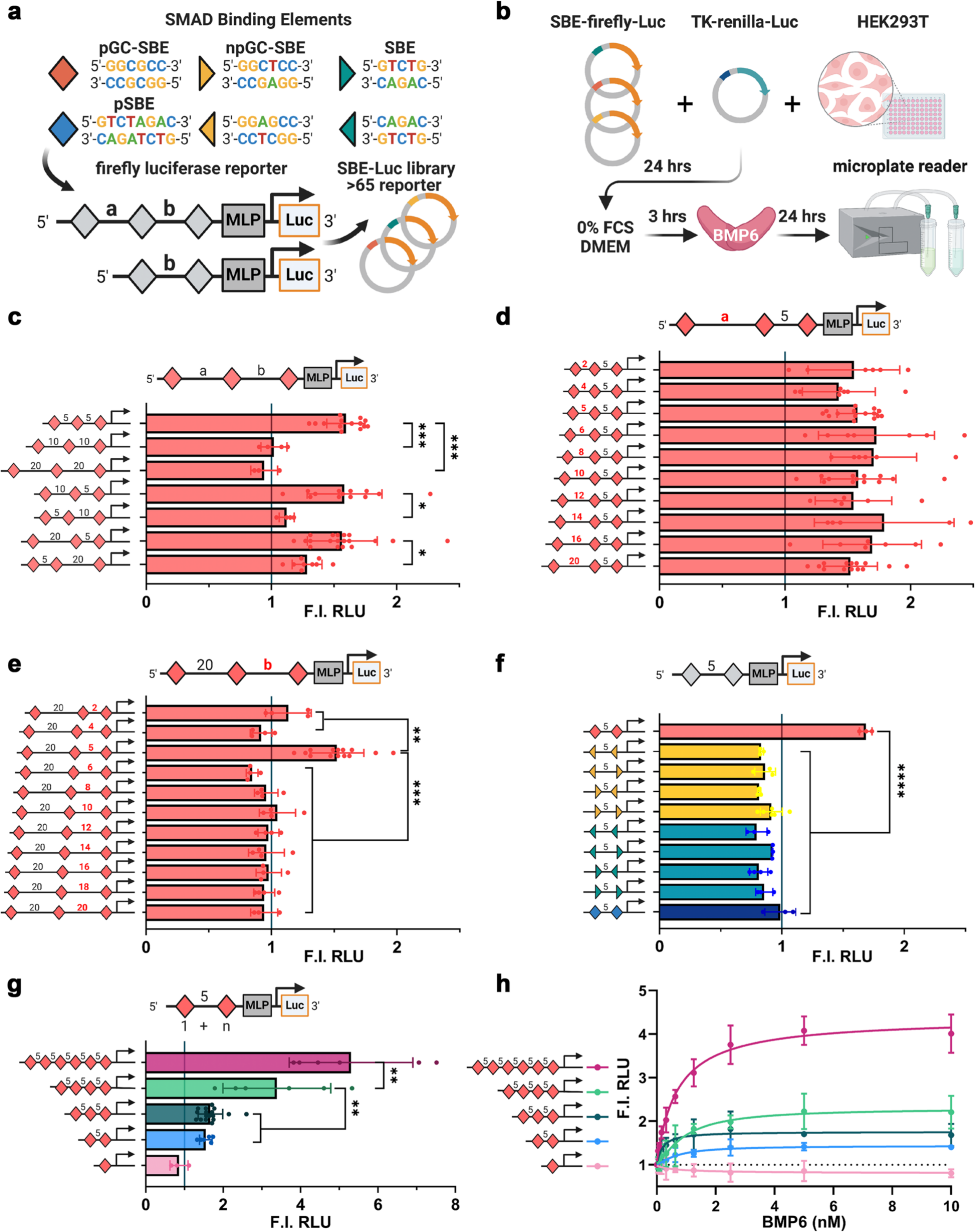
5-bp spaced pGC-SBE homocomposite motifs encode BMP responsiveness: **a** A library of synthetic firefly luciferase construct was cloned with 1 to 6 SMAD motifs positioned 10 bp before a minimal promotor (MLP) with varying spacer length for pGC-SBEs and varying orientation for npGC-SBE and SBE motifs. **b** SBE-firefly-Luc constructs were co-transfected with TK-renilla luciferase in HEK293t cells; cells were starved and stimulated with BMP6 (5 nM) for 24 h before analysis using a microplate reader (**c**–**h**). **c**–**e** Dual luciferase reporter assay displaying BMP6 responsiveness towards constructs with differently spaced pGC-SBE homocomposite motifs. BMP6 responsiveness is observed, when at least two pGC-SBEs are separated by a 5-bp spacer. **f** Dual luciferase reporter assay displaying BMP6 responsiveness towards constructs with different 5-bp spaced homocomposite motifs taking all potential orientations of non-palindromic motifs into account. 5-bp spaced npGC-SBE, pSBE, and SBE homocomposite motifs show no BMP6 responsiveness compared to pGC-SBE homocomposite motifs. **c**, **g**–**h** Dual luciferase reporter assay displaying BMP6 responsiveness towards constructs with one to six 5-bp spaced pGC-SBE homocomposite motifs (5 nM (**g**) or 0.04 to 10 nM BMP6 (**h**)). **c**–**h** Data are shown as mean fold induction to unstimulated cells (gray line) in relative luciferase units (RLU) ± SD (**c**–**g** *n* = 3–15; **h** *n* = 3 independent experiments). Statistical significance was calculated in between samples using one-way ANOVA and Tukey’s post hoc test; *P < 0.05, **P < 0.01, ***P < 0.001, and ****P < 0.0001

Since all MH1 domains can bind to single SMAD motifs, an important question arises: what are the DNA sequence requirements for the efficient binding of dimeric/trimeric SMAD1/5/8 complexes that subsequently enhance target gene transcription? To address this question, we constructed a library comprising > 65 firefly luciferase constructs. These constructs contain a minimal promoter (major late promoter—MLP) preceded (10 bp distance) by up to six SMAD motifs with varying sequences, spacing, and orientations (Fig. 2a).

First, we determined the optimal concentrations of BMP6 (5 nM) or TGFβ1 (0.2 nM) required to induce a robust signal response. This was achieved by performing dual luciferase assays with BRE_2_-luc [17] and CAGA_12_-luc [15] constructs, followed by serial dilution of ligands (Additional file 1: Fig. S2).

Next, we tested a series of luciferase constructs containing three pGC-SBE motifs with varying spacing between motif 1 and 2 (designated as linker a) and between motif 2 and 3 (designated as linker b), using GC-poor linker sequences (Fig. 2b–e). Only constructs with at least one pair of motifs spaced by 5 bp showed sensitivity to BMP6, while all the other spacings tested (ranging from 2 to 20 bp) remained unresponsive to BMP6 stimulation (Fig. 2c–e). Notably, this response was significantly reduced when the 5-bp spaced composite motif was positioned further upstream of the MLP (linker b = 10/20 bp), underscoring the critical impact of the relative distance between the SMAD composite motif and the MLP (Fig. 2c, Additional file 1: Fig. S3a). In addition, variations in the spacing of the third binding motif did not influence BMP6 sensitivity when a 5-bp spaced pGC-SBE motif pair was present (Fig. 2d). Furthermore, deletion or addition of 1 bp to the linker abolished signaling (Fig. 2e). EMSA experiments using npGC-SBE homocomposite motif oligos show that SMAD1-MH1 domain double binding is not inhibited by linker lengths below 5 bp (Additional file 1: Fig. S4), suggesting that the full-length SMAD complex is defining the 5 bp selectivity.

Next, we demonstrated that a homotypic set of two pGC-SBE motifs alone is sufficient for mediating BMP6 sensitivity while all other possible combinations of homotypic 5-bp spaced npGC-SBEs, pSBE, or SBEs did not induce signaling (Fig. 2f). Remarkably, none of the aforementioned constructs showed sensitivity to TGFβ1 stimulation (Additional file 1: Fig. S5), except the homotypic combination of two 5-bp spaced pSBE motifs (Additional file 1: Fig. S5f), thereby pointing to a specific binding mode for SMAD1/5/8. Finally, the presence of additional 5-bp spaced pGC-SBE motifs (4 and 6) further enhanced BMP6 responsiveness and yielded reporter constructs that are highly sensitive at low BMP concentrations (Fig. 2g–h).

Taken together, these findings underscore that clustering of GC-rich SMAD motifs alone does not suffice to convey BMP sensitivity. These results thus highlight the critical importance of the relative distance between motifs and other TF motifs within the MLP.

### Orientation of the motifs within the heterocomposite motif determines SMAD binding preferences

**Fig. 3:**
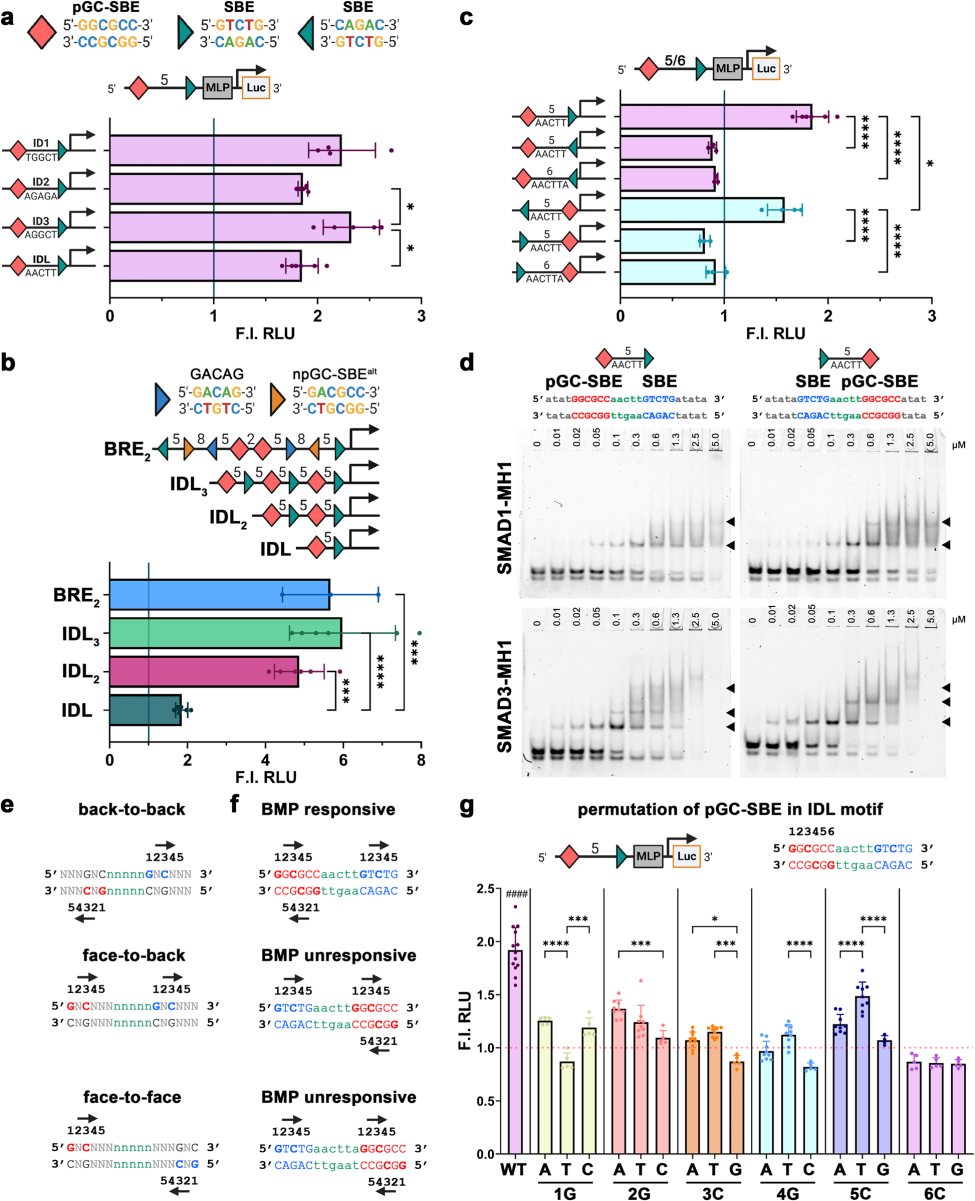
5-bp spaced pGC-SBE/SBE heterocomposite motifs reveal preferred MH1 binding orientation: **a**–**c**, **g** Dual luciferase reporter assay displaying BMP6 responsiveness towards constructs with pGC-SBE/SBE heterocomposite motifs. Whereas composite motifs with different linker sequences originating from ID1/2/3 loci compared to a control (IDL—ID-like) GC-poor linker are all BMP6-responsive (**a**), the relative orientation of the non-palindromic SBE motif within the composite is indicative of BMP6 responsiveness (**c**). BMP6 responsiveness increases with multiplication of the ID-like (IDL) composite motif to a similar level (IDL_3_) of the BRE_2_ reporter construct (**b**). Data are shown as mean fold induction to unstimulated cells (gray line) in relative luciferase units (RLU) ± SD (*n* = 3–6 independent experiments). Statistical significance was calculated in between samples using one-way ANOVA and Tukey’s post hoc test. **d** EMSA experiments were performed testing the binding of human SMAD1/3-MH1 domains to differently oriented pGC-SBE/SBE heterocomposite motifs. Protein concentrations (µM) are shown on top of the EMSA. Abbreviations for the DNA oligonucleotides and dsDNA sequence are shown above. Single, double, and triple SMAD-MH1 binding to dsDNA is indicated with black triangles. **e**–**f** Schemes indicating binding mode of two SMAD-MH1 domains towards composite motif. Black arrow shows SMAD-MH1 binding based on the first bound Gua (bold). **e** Theoretically SMAD-MH1 domains could bind in face-to-face, face-to-back, and back-to-back orientation. **f** Sequence comparison of BMP-responsive and unresponsive heterocomposite motifs with indicated SMAD-MH1 binding orientation. **g** Dual luciferase reporter assay displaying BMP6 responsiveness towards constructs containing permutated pGC-SBE/SBE heterocomposite motifs with (CTRL) GC-poor linker. While all variations lead to a decrease in responsiveness compared to WT, 6th C/1st reverse G is the most essential. Data are shown as mean fold induction to unstimulated cells in relative luciferase units (RLU) ± SD (*n* = 5–13 independent experiments). Statistical significance was calculated in between samples (*) or relative to WT ctrl (#) using one-way ANOVA and Šídák’s (*) or Dunnett’s (#) multiple comparisons test; *P < 0.05, **P < 0.01, ***P < 0.001, and ****/####*P* < 0.0001

A heterocomposite motif consisting of pGC-SBE and SBE motifs, separated by 5 bp, has been described as BMP-sensitive and is located in SMAD1-bound regions of canonical BMP-target genes like *ID1/2/3* [16, 17]. To ascertain whether these naturally occurring “*ID*-type” motifs (GGCGCC-N_5_-GTCTG) convey BMP sensitivity in vitro, we compared the effect of “*ID native* linkers” (*ID1* TGGCT; *ID2* AGAGA; *ID3* AGGCT) with a synthetic GC-poor linker (AACTT) (Fig. 3a). Intriguingly, all four constructs displayed BMP sensitivity, with the ID1 and ID3 constructs containing a GC-rich linker showing a stronger response. Similarly, multiplying the ID-like motif (ID-like reporter, IDL) two or three times further increases BMP sensitivity to a degree comparable to the BRE_2_ (Fig. 3b, Additional file 1: Fig. S3b).

Given that the palindromic GC-SBE motif is the same on both the sense and antisense strands, we inverted the SBE motif to study whether the relative orientation is crucial for BMP sensitivity (Fig. 3c). Surprisingly, BMP sensitivity was lost when the SBE motif was located on the antisense strand (GGCGCC-N_5_-CAGAC) and this sensitivity could not be restored, even by adjusting the linker to match the composite motif length of the homotypic pGC-SBE composite motif (6 + 5 + 6 bp). However, the heterocomposite motif on the antisense strand (CAGAC-N5-GGCGCC) maintained BMP sensitivity, albeit to a lesser extent than when located on the sense strand (Fig. 3c, Additional file 1: Fig. S3c). We performed parallel EMSAs with SMAD1 or SMAD3 MH1 domains and tested for shifts indicative of double MH1 binding (Fig. 3d). Interestingly, both heterocomposite motifs showed similar SMAD1/3 binding irrespective of SBE orientation (Fig. 3d). This observation suggests that the effect of the orientation and linker length on BMP sensitivity is observable only when using full-length proteins capable of associating through their MH2 domains and not when using isolated MH1 domains.

Given the conserved binding mode of all SMAD MH1s, favoring a Gua at the + 1 and a Cyt at the + 3 position within their binding motifs [19], we explored the potential binding orientations of two SMAD MH1 domains within a composite motif (Fig. 3e). While all double binding modes are theoretically possible with the homotypic pGC-SBE double motif, the “ID-like” heterotypic composite motif would only allow a face-to-back or back-to-back binding mode, due to the fixed orientation of MH1 binding to the SBE motif (Fig. 3f).

To identify the correct binding orientation of SMAD MH1 within the palindromic GC-SBE of the “ID-like” motif, we permutated each nucleotide and compared the activity of the resulting heterocomposite motifs (Fig. 3g). These constructs also contained the non-palindromic GC-SBE motif (GGCTCC) in different orientations. Notably, any substitution of a nucleotide within pGC-SBE led to a significant reduction or loss of BMP sensitivity, including both orientations of npGC-SBE (+ 3 position Cyt to Ade; + 4 position Gua to Thy) (Fig. 3g). Permutating the C at the + 6 position completely abrogated BMP sensitivity, while mutating the Gua in the + 1 position (to Ade and Cyt) maintained reduced responsiveness. These data suggest that within both a 5-bp spaced homotypic and heterotypic SMAD composite motif, a functional BMP-sensitive SMAD complex can bind only in a back-to-back mode, with the two Gua residues in + 1 position adjacent to the linker sequence on opposing strands. When considering the 3D structure of DNA, the twist of the double-stranded helix brings the Gua bases close together, facilitating the binding of a SMAD complex from one direction. Finally, none of the heterocomposite motifs showed sensitivity to TGFβ1 stimulation (Additional file 1: Fig. S6), and all luciferase reporter findings were reproducible using U2OS cells (Additional file 1: Fig. S7), thus demonstrating transferability to different cell types.

### Identification of SMAD composite motifs enhances predictive value of BMP responsiveness

**Fig. 4:**
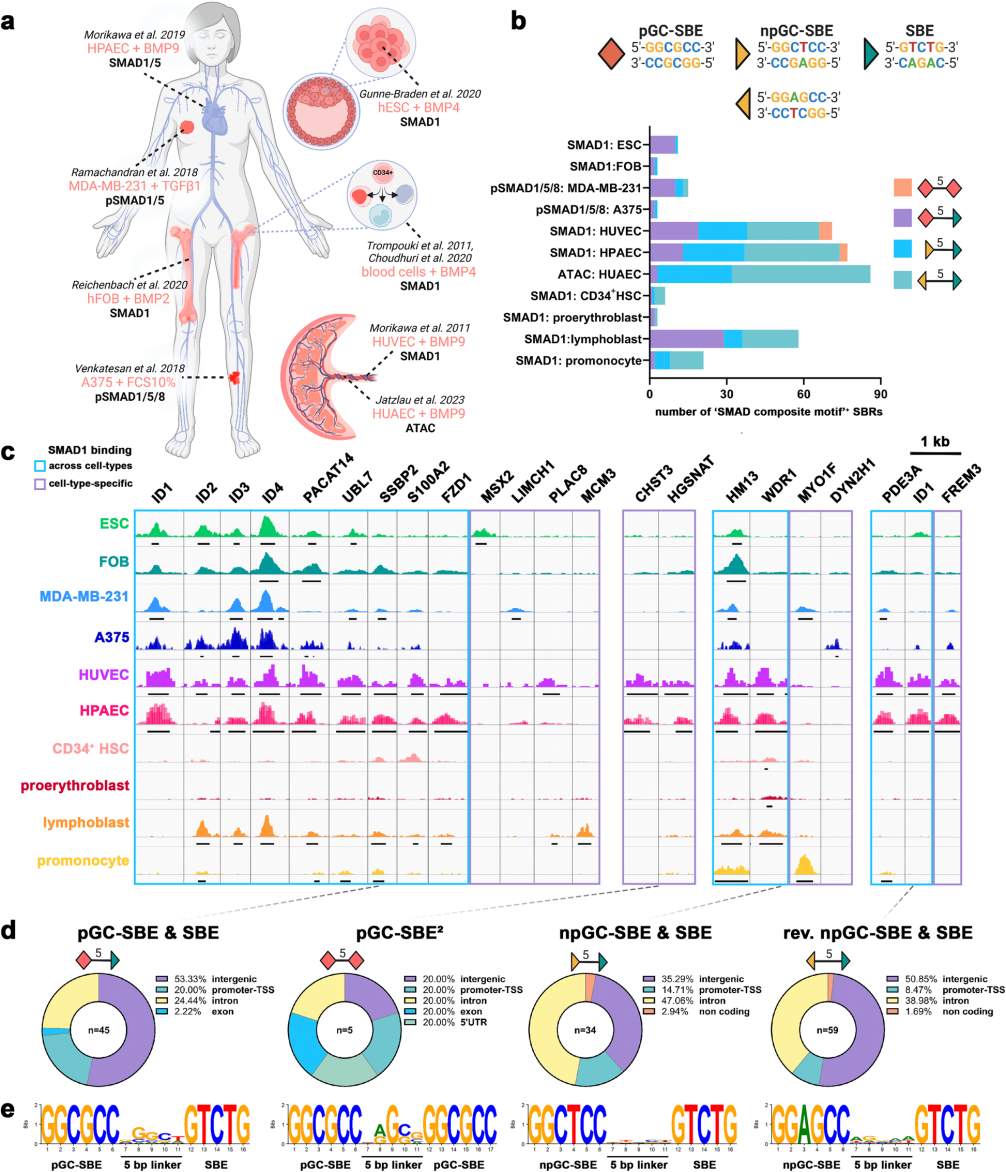
5-bp spaced SMAD motifs are active across different tissues: **a** Schematic overview of publicly available SMAD1, SMAD1/5, or pSMAD1/5 ChIP-Seq datasets and ATAC-Seq data from a variety of cell types and stimulations extracted from ChIP-Atlas (52). **b **Absolute number of SMAD1-bound regions positive for 5-bp spaced SMAD homo-/heterocomposite motifs. **c** Genome browser view highlighting selected SMAD1-bound regions positive for SMAD composite motifs. **d** Percentage distribution of “SMAD composite motif”-positive SMAD1-bound regions in functional genomic regions according to Homer gene annotation. Percentages are normalized over the genomic abundance of each functional region. **e** Position weight matrices generated with DNAlogo 4.0 using all identified SMAD composite motifs reveal enriched linker sequences

Following the delineation of the minimal functional SMAD composite motif for BMP sensitivity, we conducted a comprehensive screening to identify the presence of these composite motifs within a diverse array of publicly available datasets, including SMAD1, SMAD1/5, and pSMAD1/5 ChIP-Seq, as well as BMP9-stimulated ATAC-Seq datasets from various tissues (Fig. 4a, Additional file 2: Table 1) [13, 16, 29–34]. Our analysis revealed the presence of the “ID-like” heterocomposite SMAD motif in 45 distinct SBRs (SMAD-bound regions) across cell types, whereas the pGC-SBE homocomposite motif was detected in only 5 SBRs in SMAD1 ChIP-Seq of HUVECs (human umbilical vein endothelial cells) and HPAECs (human pulmonary arterial endothelial cells) [16, 29] (Fig. 4b–c). Heterocomposite motifs comprising an npGC-SBE and an SBE motif were predominantly observed in endothelial SBRs and BSRs (BMP-sensitive regions), as well as SBRs from lymphoblasts and promonocytes (Fig. 4b–c). Interestingly, the relative number of SBRs positive for 5-bp spaced SMAD composite motifs is very small compared to the total amount of SBRs (e.g., HPAECs: 5-bp comp motif^+^*n* = 62 ≙ 0.003%, total SBR *n* = 17.301).

Notably, “ID-like” heterocomposite SMAD motifs were detected in SBRs present across different experimental approaches and cell types, including those proximal to *ID1/2/3/4*, *PACAT14*, *UBL7*, and *SSPB2* loci, as well as in cell type-specific SBRs such as *MSX2* in ESCs (embryonic stem cells) or *LIMCH1* in MDA-MB-231 cells (Fig. 4c). SBRs harboring “SMAD-composite motifs” were predominantly located in intergenic enhancers, with fewer occurrences in intronic enhancers or promoter regions (Fig. 4d).

Furthermore, we compared all SMAD1-bound composite motifs to identify a preferential linker sequence, revealing enrichment for Gua at the + 2 and/or + 3 position of the 5 bp linker (Fig. 4e). This finding is consistent with the higher BMP sensitivity observed in ID1- and ID3-Luc reporters, both featuring a Gua at the + 3 position, compared to our GC-poor linker (Fig. 3a). In summary, our results underscore the presence of BMP-sensitive SMAD composite motifs within a minority of SMAD1-bound regions across diverse tissues (Additional file 2: Table 1), thereby emphasizing the significance of motif and linker sequence, as well as the relative orientation and spacing of SMAD motifs.

## Discussion

SMAD-mediated transcription involves complex organization, with specificity not solely determined by a single MH1 target site recognition event, but instead dependent on the concerted binding of two MH1 domains within a SMAD dimer/trimer to a SMAD composite motif. Our study highlights that the specificity of BMP and TGFβ-dependent target gene regulation cannot be explained by preferential binding of single SMAD-MH1 domains to SMAD binding elements (SBE, pGC-SBE, and npGC-SBE), as R-SMAD1 and 3 and co-SMAD4 MH1 domains all bound to these motifs. However, responsiveness specific to BMP-sensitive SMADs was strongly dependent on the presence of at least two SBEs with specific distance and orientation. These observations thus underscore the importance of exploring the boundaries of SMAD binding towards composite motifs.

### Affinities of BMP and TGFβ SMADs to single binding elements

Using isothermal titration calorimetry (ITC), SMAD4 MH1 was previously determined to bind SBE (*K*_d_ of 160.3 ± 0.2 nM) and 5-bp GC-SBE (GGCGC; 270.5 ± 0.1 nM) containing oligos with a mid-nanomolar affinity [19]. This is similar to our findings as we estimated the binding affinity from EMSAs for SMAD1/3/4 to pGC-SBE, npGC-SBE, and SBE equally in the mid-nanomolar range (100–1000 nM). However binding to pGC-SBE was observed with higher affinity for all three SMAD MH1s compared to npGC-SBE, in line with previous reports that have observed the same for SMAD1-MH1 [16]. This difference is also reflected on the level of reporter gene activity, where exchanging a single nucleotide of the palindromic GC-SBE nearly completely abolishes BMP responsiveness in all tested non-palindromic GC-SBE reporters.

Our comparison between SMAD1 and SMAD3-MH1 did show similar affinities to pGC-SBE and a slightly higher affinity of SMAD3-MH1 to SBE, suggesting no big differences in the recognition of single motifs. However, a previous study has focused on comparing the binding mode of SMAD1-MH1 and SMAD3-MH1 to single palindromic binding sites, including pSBE or pGC-SBE [20], and concluded a higher cooperative binding mode for SMAD1-MH1 to these motifs. In line with their work, we equally found double binding of SMAD3-MH1 to pGC-SBE with only a moderate positive cooperativity. However, we did not observe cooperative binding for SMAD1-MH1, which can be explained by a GC-free flanking sequence in our oligos, which is different to the design used in the previous study and should prevent unintended secondary binding events. Furthermore, it has to be considered that SMAD1-MH1s can also dimerize via their N-terminus [35], making a clear discrimination between true double binding and binding of an SMAD1-MH1-dimer impossible without further modifications.

### Composite motif syntax defines BMP response

Two models, namely the billboard and enhanceosome models, have been proposed to describe the syntax of TF binding sites within enhancer regions. While the former suggests that the presence of specific TF motifs alone is sufficient for TF binding and gene regulation [36], the latter argues for a requirement of specific order, orientation, and spacing of TF motifs within the enhancer sequence [26, 27]. Our findings align with the enhanceosome model, as demonstrated by the significance of motif order, orientation, and positioning in the regulation of SMAD-target genes. Essentially, we show that clustering of palindromic GC-SBE motifs does not convey BMP or TGFβ sensitivity unless one pair is 5-bp spaced. Further, there was no requirement of a third binding site to convey BMP sensitivity. Clustering of SMAD binding sites will rather increase the local likelihood of SMAD-binding or allow binding of multiple SMAD complexes.

Early studies on TGFβ-dependent regulation of *PAI-1* revealed the significance of repetitive AGAC motifs, leading to the development of the highly sensitive CAGA_12_-luc construct [15, 37]. It was further shown that the orientation of SBEs in homotypic or tripartite composite motifs defines the responsiveness to SMAD3/4, a feature attributed to steric limitations imposed by MH2 oligomerization [38]. Similarly, the mirrored duplication of SBE and GC-SBE motifs in the *ID1* promoter established the BMP-specific BRE_2_-luc reporter [17]. These findings underscore the importance of motif order, orientation, and positioning in the regulation of SMAD-target genes. Our study further elucidates that the relative orientation of non-palindromic heterocomposite motifs crucially determines responsiveness to BMP stimulation. Additionally, we highlight the influence of motif positioning and distance, emphasizing that any deviation from a 5-bp spacer length compromises the responsiveness of composite motifs.

This observation aligns closely with findings previously reported in *Drosophila melanogaster*, where the relative 5-bp spacing in the Dpp-sensitive silencer element (SE, GNCGNC(N)_5_GTCT) was not detrimental for binding of SMAD homologs Mad/Medea, but for recruitment of the corepressor Schnurri and repression of Dpp-target genes [39, 40]. In contrast, the linker length of the very similar activating element (AE, GGCGCCA(N)_4_GNCV) composite motif was reported to be more flexible [40, 41].

### Differential responses of SMAD1/5 and SMAD2/3 complexes to spaced motifs

**Fig. 5:**
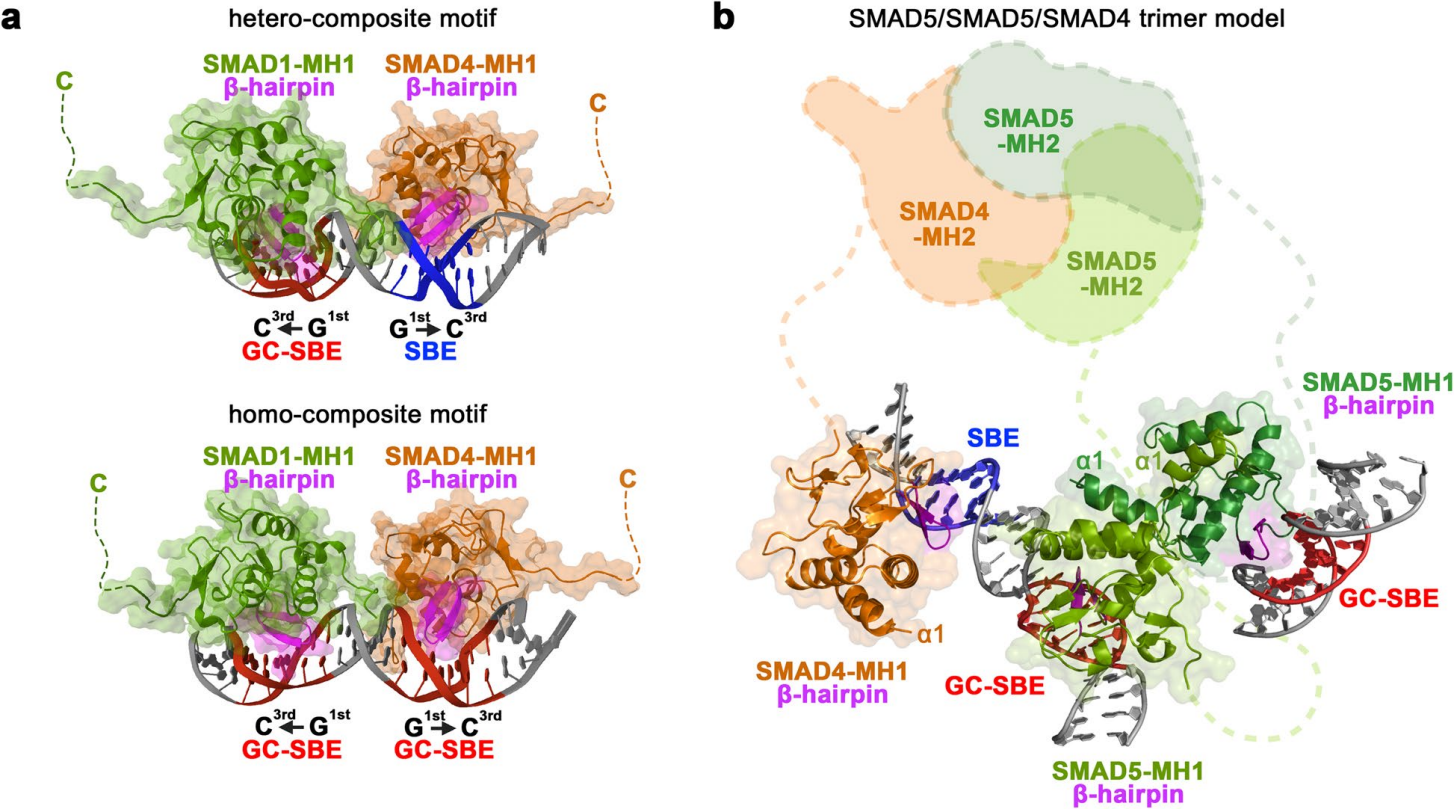
Back-to-back binding of BMP-sensitive SMADs: **a** SMAD-MH1 docked to GC-SBE/SBE and GC-SBE/GC-SBE composite motifs in back-to-back binding mode. As both SMAD1 and SMAD4-MH1 bind pGC-SBE and SBE motifs, the shown binding mode is just one representative model. It is equally likely that SMAD4-MH1 binds to the pGC-SBE and SMAD1-MH1 to the SBE motif. **b** Model of a trimeric SMAD5/5/4 complex binding to an ID-like composite motif on one dsDNA strand and a second GC-SBE motif at another locus. SMAD5-MH1 (dimer, green, PDB: 6FZS) and the SMAD4-MH1 (orange, PDB: 5MEY) were superimposed to docked SMAD1-MH1 and SMAD4-MH1 from **a**. The view highlights that the limiting factor determining binding to target motifs of BMP-sensitive SMAD complexes must be the structure of the dimeric/trimeric MH2 domains

Our results indicate that in human cells a minimal BMP-SMAD response requires two SMAD motifs with each initial Gua nucleobase of the motifs adjacent to the spacer in a mirrored fashion, leading to a back-to-back binding of two SMAD-MH1 domains. This finding emphasizes the importance of SMAD motif architecture within enhancers in defining BMP pathway-specific target gene regulation. Docking of SMAD1-MH1 and SMAD4-MH1 to either GC-SBE homocomposite or “ID like” heterocomposite motif highlights that the back-to-back binding mode is possible without steric hindrance of the MH1 domains (Fig. 5a), suggesting that the limitation in binding is dictated by the full-length structure of the dimeric/trimeric BMP-sensitive SMAD complex (Fig. 5b). A key difference in SMAD1/5/8 vs SMAD2/3 complex binding is the preferential spacing of 5 bp specific for SMAD1/5/8, which is more flexible for SMAD2/3 [28]. One possible explanation for this observation is the propensity of SMAD1/5/8-MH1 domains to form helix-swapped dimers, which may hinder the selection of palindromic 3-nucleotide binding sites compared to adjacent or distant binding sites [35]. In contrast, two SMAD2/3/4-MH1 domains can bind to the same pSBE (GTCTAGAC) motif [42].

Another key mechanism that governs transcriptional activation downstream of R-SMAD DNA binding is the recruitment of co-TFs. As demonstrated in *Drosophila* with Mad/Medea and Schnurri [39, 40], it is possible that all SMADs can bind due to their relatively flexible and long linker regions. However, only those complexes that bind in a specific arrangement conducive to recruiting transcriptional modulators are transcriptionally active.

Even though both SMAD1-MH1 and SMAD3-MH1 bound all tested SMAD motifs, no TGFβ1 response was observed for the BMP-sensitive homotypic pGC-SBE reporter or the IDL reporters. In contrast only the 5-bp spaced homotypic pSBE composite motif showed TGFβ1 responsiveness. This is in line with a study demonstrating high TGFβ1 responsiveness of a 3-bp spaced dual pSBE reporter (SS-Luc) [28]. The authors further show that either one pSBE (GTCTAGAC) or a triple SBE (GTCTG) motif forms a *cis*-acting functional half-unit, with two half-units required for efficient SMAD2/3-dependent transcription activation [28]. They propose that binding of two activated SMAD2/3 complexes to such a cis regulatory element would facilitate SMAD-bound p300 dimerization, which thereby gets activated [43]. Collectively, this highlights that BMP and TGFβ target gene specificity is not defined by single SMAD binding motifs, but rather the arrangement of at least two motifs in a composite motif, which will select for a specific SMAD complex bound by transcriptional effectors.

### Context-dependent recruitment of SMAD1/5 complexes

SMAD1 binding mostly occurs at enhancer regions across various cell types (Fig. 4) [16]. Our analysis reveals an intriguing observation regarding the prevalence of BMP-sensitive 5-bp spaced composite motifs within SMAD-bound regions across various tissues. Surprisingly, the absolute number of such motifs is relatively low (0.003% of all SBRs in HPAECs), indicating that SMAD1 binding is likely mediated by composite motifs with a different architectural arrangement in most cases. Consistent with the outcomes of our reporter studies, we have previously identified SMAD composite motifs with linkers longer than 5 bp in proximity to genes that require high levels of phosphorylated SMAD1/5 for transcription (referred to as “pSMAD1/5 high dose targets”) [13]. Given that SMAD-binding entails a low-affinity interaction of each MH1 domain with its targets, the recruitment of SMADs to specific genomic loci is likely facilitated by context-specific co-transcription factors, leading to localized recruitment or stabilization of SMAD-binding and subsequent transcriptional activation [6, 44]. The relevance of the *cis*-environment of BMP-response elements for tissue specificity and dose sensitivity was equally shown in *Drosophila melanogaster* [41]. Notably, our observations align with previous reports indicating the presence of motifs associated with GATA and SOX family TFs in proximity to sub-optimal SMAD composite motifs [13], corroborating documented interactions between GATA and SOX TFs and SMADs [45, 46]. The recruitment of low-affinity SMADs by co-transcription factors underscores the highly cell-type and context-dependent nature of BMP signaling [11].

## Conclusions

In summary, our findings shed light on the mode of SMAD1/5/8-DNA binding and transcription initiation, providing insights into the regulatory mechanisms governing BMP-sensitive SMAD target gene expression. Understanding these mechanisms not only elucidates key steps in embryonic development [33] but also offers valuable insights into disease-associated contexts [13]. Furthermore, our discoveries hold promise for the development of novel synthetic enhancers with potential clinical applications [35].

## Methods

### Protein expression and purification

SMAD1 MH1 (Uniprot: P70340), SMAD3 MH1 (Uniprot: P84022), and SMAD4 MH1 (Uniprot: Q13485) domain proteins were cloned in pOPINSF using synthesized DNA templates (Thermo Fisher Scientific). All clones were confirmed by DNA sequencing. All protein constructs were expressed fused to an N-terminal His-tag followed by a TEV or 3C cleavage sites in *Escherichia coli* B834(DE3) strain and purified following standard procedures [19, 35]. Cells were grown at 37°C to reach an OD_600_ of 0.6 and induced with IPTG (0.4 mM) followed by overnight expression at 20°C. Bacterial cultures were centrifuged, and cells were lysed at 4°C using EmulsiFlex-C5 (Avestin) in the presence of lysozyme. Supernatants containing the soluble proteins were purified by nickel-affinity chromatography (HiTrap Chelating HP column, GE Healthcare Life Science) using NGC Quest 10 Plus Chromatography System (BIO-RAD). Eluted proteins were cleaved with TEV or 3C proteases (at 4°C or room temperature, respectively), and further purified by ion exchange chromatography (HiTrap SP HP column, GE HealthCare). Finally, all MH1 domains were purified by size-exclusion chromatography on a HiLoad™ 16/600 Superdex™ 75 pg (GE Healthcare Life Sciences) in 20 mM Tris, 80 mM NaCl, and 2 mM TCEP at pH 7.2 and kept at 80°C. Purified proteins were verified by MALDI-TOF mass spectrometry.

### Electrophoretic mobility shift assay (EMSA)

Short duplex Cy®5-DNA was annealed using complementary single-strand HPLC purified DNA. Complementary strands were mixed at equimolar concentrations (2 mM) in 10 mM Tris pH 7.2 at 25°C and heated at 95°C for 5 min and cooled to room temperature for 2 h. Protein-DNA binding reactions were carried out for 15 min on ice in 10 μL of binding buffer (200 mM TRIS pH 7.2, 100 mM NaCl, and 2 mM TCEP). A fixed concentration of 5′-end Cy5-labeled (Biomers, Germany) duplex DNA (7.5 nM) was incubated with increasing amounts of SMAD1 MH1, SMAD3 MH1, SMAD4 MH1, and SMAD5 MH1 purified proteins, and then 10 µL of Orange G Loading Dye 2X was added to the mixture. Binding reactions were loaded into 6% polyacrylamide gels (1.5 mm thick), prepared with 19:1 40% acrylamide solution (PanReac AppliChem). The gels were run for 1 h in TG buffer at 100 V at 4°C. Fluorescent images of the gels were acquired with the Cy5 channel on a using a ThyphoonTM 6800 (Molecular Dynamics).

The data of two independently performed replicates were fit to the Hill equation: *Y* = Bmax * *X*^*h*/(*K*_d_^*h* + *X*^*h*) using PRISM, where *Y* is the response, Bmax is the maximum response (Bmax = 0–1), *X* is the ligand concentration, *K*_d_ is the dissociation constant (*K*_d_ > 0), and *h* is the Hill coefficient (*h* > 0). Nonlinear regression was performed to estimate the parameters Bmax, *K*_d_, and *h*.

### Docking and MD simulation

For this study, we began by preparing the SMAD MH1-DNA complexes using a combination of advanced computational tools. To create the DNA structures, we used Chimera’s built-in tool for designing nucleic acids, ensuring that the DNA sequences were accurately represented for our simulations. Once the structures of both the protein and DNA were ready, we used HDOCK [47, 48], a software package, to dock the SMAD MH1 protein to the DNA sequences. This step was crucial as it helped us predict how the protein and DNA would interact, forming the initial complexes that were to be simulated. We conducted simulations on SMAD MH1-DNA complexes, employing the GROMACS 2021 software suite [49] for a detailed analysis over a span of 10 ns. The simulations used the AMBER99SB-ILDN force field [50] for protein interactions and AMBER94 for nucleic acid [51] behavior to accurately represent the complex molecular interactions. The SPC/E (Simple Point Charge/Extended) water model was adopted to solvate the complexes, ensuring a realistic environmental setting for the protein-DNA systems. Initial system minimization was undertaken using the steepest descent method within GROMACS, targeting a reduction of the maximum force (Fmax) to below 100.0 kJ/mol/nm, thereby preparing the complexes for subsequent equilibration phases. The equilibration process was divided into two distinct steps. Initially, the NVT ensemble (constant number of particles, volume, and temperature) was applied for 1 ns or 500,000 steps to achieve temperature stability within the complexes. This was followed by the NPT ensemble (constant number of particles, pressure, and temperature) for an additional nanosecond or 500,000 steps, aimed at ensuring both pressure and temperature equilibrium across the system. Following the successful equilibration, the production phase of molecular dynamics (MD) simulation was conducted with a 2 fs timestep, capturing the dynamic interactions and structural changes of the SMAD MH1-DNA complexes over time. After completion of all simulation stages, the results were thoroughly analyzed using GROMACS 2021, and a representative energy optimized SMAD-DNA complex was represented in pymol. Finally, to model a trimeric SMAD5/5/4 complex bound to three SBEs, the crystal structures of SMAD5-MH1 (dimer, green, PDB: 6FZS) and SMAD4-MH1 (orange, PDB: 5MEY) were superimposed to the β-hairpin of docked SMAD1-MH1 and SMAD4-MH1 in pymol.

### Cell culture

HEK293T and U2OS cells were obtained from the German Collection of Microorganisms and Cell Cultures (DSMZ) and cultured in Dulbecco’s Modified Eagle’s Medium (DMEM) supplemented with 10% FCS, 2 mM L-glutamine, and penicillin (100 units/mL)/streptomycin (100 µg/mL) (DMEM full medium) in a humidified atmosphere at 37°C and 5% CO_2_ (v/v).

### Cell stimulation with growth factors

rhBMP6 (gift from S. Vukicevic, Univ. of Zagreb, Croatia) and rhTGFβ1 (PeproTech, Hamburg, Germany) were reconstituted and stored following the manufacturer’s instructions. For stimulation, these growth factors were added to the cells with the indicated concentrations in DMEM following serum starvation.

### Cloning of luciferase reporter constructs

A minimal adenoviral major late promoter (MLP) (GGGCTATAAAAGGGGGTGGGGGCGCGTTCGTCCTCACTCTCTTCC) fragment was subcloned from the BRE_2_-luc [17] between BglII and HindIII sites upstream of the luciferase gene in the pGL3-basic vector. Minimal SMAD composite motifs contained 1 to 6 pGC-SBE (GGCGCCC), npGC-SBE (GGCTCC), SBE (GTCTG), or pSBE (GTCTAGAC) motifs in varying distances and orientations, with GC-poor spacers up to 20 bp, if not otherwise stated. To generate a firefly luciferase reporter library of 65 different constructs (Additional file 3: Table 2) carrying defined BMP-activated minimal SMAD composite motifs, forward and reverse oligonucleotides were annealed using 1 × T4 ligation buffer at 95°C and then phosphorylated with the T4 polynucleotide kinase. The annealed and phosphorylated oligos were then ligated using T4 ligase into the pGL3-basic MLP vector using KpnI and XhoI restriction sites upstream of the MLP. The resulting distance of the first SMAD motif to the MLP is 10 bp (overlapping XhoI and BglII sites). The ligation was inactivated at 65°C for 10 min and the construct transformed using chemically competent DH5α *E. coli* bacteria. Plasmid DNA purification was performed using NucleoSpin Plasmid EasyPure Kit following the manufacturer’s guidelines, followed by a Sanger sequencing.

### Dual luciferase reporter gene assay

HEK293T cells were transfected using polyethylenimine (PEI) with either BRE_2_-luc, CAGA_12_-luc, or Luciferase reporter constructs carrying synthetic SBE composite motifs. A constitutively expressing construct encoding renilla luciferase (RL-TK; Promega) was co-transfected as internal control. The next day, cells were starved in serum-free medium for 3 h and then stimulated with 5 nM BMP6 or 0.2 nM TGFβ1 for 24 h. Cell lysis was performed using passive lysis buffer (Promega) and luciferase activity was measured with a TECAN Spark Luminometer, following the manufacturer’s instructions.

### Analysis of SMAD motif occurrence in sequencing data

Publicly available sequencing data (ChIP-Seq, ATAC-Seq) were downloaded either from www.chip-atlas.org [52] or GEO database and are listed in Additional file 2: Table 1. We constructed HOMER [53] motif libraries for single SMAD binding elements or composite motifs (2 motifs spaced by 5 random selected nucleotides (N_5_)). HOMER’s annotatepeaks.pl script with default parameters (hg38) was used to count the occurrence of single or composite motifs in peak bed files.

### Statistical analysis

Statistical tests were performed using GraphPad Prism (v9.3) software. All statistical tests are listed in the figure legends. Normal distributions of data sets were tested with the Shapiro–Wilk normality test. In cases of failure to reject the null hypothesis, the ANOVA and Bonferroni post hoc test were used to check for statistical significance under the normality assumption. Data points normalized to 1 were not statistically compared due to lack of a normal distribution. For all experiments statistical significance was assigned, with an alpha level of *P* < 0.05.

## Supplementary Information

Additional file 1: Supplementary Figs. S1–S7. Fig. S1 SMAD-MH1 purity control and experimental set-up. Fig. S2 BRE_2_-Luc and CAGA_12_-Luc define experimental conditions for selective comparison of BMP6 and TGFβ1. Fig. S3 BMP6 dose–response curves of SMAD composite motif reporters. Fig. S4 Composite motif spacers below 5 bp do not inhibit SMAD1-MH1 double binding. Fig. S5 BMP-responsive SMAD homocomposite motif reporters are unresponsive to TGFβ1. Fig. S6 BMP-responsive SMAD heterocomposite motif reporters are unresponsive to TGFβ1. Fig. S7 BMP specificity of SMAD composite motif reporters is maintained in U2OS cells.

Additional file 2: Table S1 SMAD1-bound peaks positive for SMAD-composite motifs.

Additional file 3: Table S2 DNA oligo sequences.

## Data Availability

All data generated or analysed during this study are included in this published article, its supplementary information files and publicly available repositories. Publicly available sequencing data, which were analysed in this study, are listed in Additional file 2: Table 1. The data supporting the findings of this study are available from the corresponding author upon request.

## Abbreviations

SMAD: Sons of mothers against decapentaplegic
BMP: Bone/body morphogenetic protein
TF: Transcription factors
TGFβ: Transforming growth factor β
CREs: Cis-regulatory elements
SBE: SMAD-binding element
pSBE: Palindromic GC-rich SBE
npSBE: Non-palindromic GC-rich SBE
SBR: SMAD-bound regions
EMSA: Electrophoretic mobility shift assay
ITC: Isothermal titration calorimetry
MLP: Major late promoter
ID: Inhibitor of differentiation
IDL: ID-like reporter
HUVECs: Human umbilical vein endothelial cells
HPAECs: Human pulmonary arterial endothelial cells
BSR: BMP-sensitive region
ESC: Embryonic stem cells
SPC/E: Simple Point Charge/Extended
DMEM: Dulbecco’s Modified Eagle’s Medium
PEI: Polyethylenimine

## References

[1] Jia S, Meng A. TGFbeta family signaling and development. Development. 2021;148(5):dev188490. 33712443 10.1242/dev.188490

[2] Cunha SI, Magnusson PU, Dejana E, Lampugnani MG. Deregulated TGF-beta/BMP signaling in vascular malformations. Circ Res. 2017;121(8):981–99. 28963191 10.1161/CIRCRESAHA.117.309930

[3] Hiepen C, Jatzlau J, Knaus P. Biomechanical stress provides a second hit in the establishment of BMP/TGFbeta-related vascular disorders. Cell Stress. 2020;4(2):44–7. 32043077 10.15698/cst2020.02.213 PMC6997947

[4] Ning J, Zhao Y, Ye Y, Yu J. Opposing roles and potential antagonistic mechanism between TGF-beta and BMP pathways: implications for cancer progression. EBioMedicine. 2019;41:702–10. 30808576 10.1016/j.ebiom.2019.02.033 PMC6442991

[5] Wu M, Chen G, Li YP. TGF-beta and BMP signaling in osteoblast, skeletal development, and bone formation, homeostasis and disease. Bone Res. 2016;4:16009. 27563484 10.1038/boneres.2016.9 PMC4985055

[6] Hill CS. Transcriptional control by the SMADs. Cold Spring Harb Perspect Biol. 2016;8(10):a022079. 27449814 10.1101/cshperspect.a022079 PMC5046698

[7] Gomes T, Martin-Malpartida P, Ruiz L, Aragon E, Cordeiro TN, Macias MJ. Conformational landscape of multidomain SMAD proteins. Comput Struct Biotechnol J. 2021;19:5210–24. 34630939 10.1016/j.csbj.2021.09.009 PMC8479633

[8] Nickel J, Mueller TD. Specification of BMP signaling. Cells. 2019;8(12):1579. 31817503 10.3390/cells8121579 PMC6953019

[9] Wu J-W, Hu M, Chai J, Seoane J, Huse M, Li C, et al. Crystal structure of a phosphorylated Smad2. Mol Cell. 2001;8(6):1277–89. 11779503 10.1016/s1097-2765(01)00421-x

[10] Lagna G, Hata A, Hemmati-Brivanlou A, Massague J. Partnership between DPC4 and SMAD proteins in TGF-beta signalling pathways. Nature. 1996;383(6603):832–6. 8893010 10.1038/383832a0

[11] Morikawa M, Koinuma D, Miyazono K, Heldin CH. Genome-wide mechanisms of Smad binding. Oncogene. 2013;32(13):1609–15. 22614010 10.1038/onc.2012.191 PMC3615190

[12] Coda DM, Patel H, Gori I, Gaarenstroom TE, Song OR, Howell M, et al. A network of transcription factors governs the dynamics of NODAL/Activin transcriptional responses. J Cell Sci. 2022;135(8):jcs259972. 35302162 10.1242/jcs.259972 PMC9080556

[13] Jatzlau J, Mendez PL, Altay A, Raaz L, Zhang Y, Mahr S, et al. Fluid shear stress-modulated chromatin accessibility reveals the mechano-dependency of endothelial SMAD1/5-mediated gene transcription. iScience. 2023;26(9):107405. 37680470 10.1016/j.isci.2023.107405 PMC10481294

[14] Liao Z, Tang S, Nozawa K, Shimada K, Ikawa M, Monsivais D, et al. Affinity-tagged SMAD1 and SMAD5 mouse lines reveal transcriptional reprogramming mechanisms during early pregnancy. bioRxiv. 2023;12:RP91434. 10.7554/eLife.91434 PMC10972565 38536963

[15] Dennler S, Itoh S, Vivien D, ten Dijke P, Huet S, Gauthier JM. Direct binding of Smad3 and Smad4 to critical TGF beta-inducible elements in the promoter of human plasminogen activator inhibitor-type 1 gene. EMBO J. 1998;17(11):3091–100. 9606191 10.1093/emboj/17.11.3091 PMC1170648

[16] Morikawa M, Koinuma D, Tsutsumi S, Vasilaki E, Kanki Y, Heldin C-H, et al. ChIP-seq reveals cell type-specific binding patterns of BMP-specific Smads and a novel binding motif. Nucleic Acids Res. 2011;39(20):8712–27. 21764776 10.1093/nar/gkr572 PMC3203580

[17] Korchynskyi O, Ten Dijke P. Identification and functional characterization of distinct critically important bone morphogenetic protein-specific response elements in the Id1 promoter. J Biol Chem. 2002;277(7):4883–91. 11729207 10.1074/jbc.M111023200

[18] Itoh F, Itoh S, Goumans MJ, Valdimarsdottir G, Iso T, Dotto GP, et al. Synergy and antagonism between Notch and BMP receptor signaling pathways in endothelial cells. EMBO J. 2004;23(3):541–51. 14739937 10.1038/sj.emboj.7600065 PMC1271801

[19] Martin-Malpartida P, Batet M, Kaczmarska Z, Freier R, Gomes T, Aragón E, et al. Structural basis for genome wide recognition of 5-bp GC motifs by SMAD transcription factors. Nature Communications. 2017;8(1):2070. 29234012 10.1038/s41467-017-02054-6 PMC5727232

[20] BabuRajendran N, Palasingam P, Narasimhan K, Sun W, Prabhakar S, Jauch R, et al. Structure of Smad1 MH1/DNA complex reveals distinctive rearrangements of BMP and TGF-beta effectors. Nucleic Acids Res. 2010;38(10):3477–88. 20147459 10.1093/nar/gkq046 PMC2879523

[21] Baburajendran N, Jauch R, Tan CY, Narasimhan K, Kolatkar PR. Structural basis for the cooperative DNA recognition by Smad4 MH1 dimers. Nucleic Acids Res. 2011;39(18):8213–22. 21724602 10.1093/nar/gkr500 PMC3185416

[22] Shi Y, Wang YF, Jayaraman L, Yang H, Massague J, Pavletich NP. Crystal structure of a Smad MH1 domain bound to DNA: insights on DNA binding in TGF-beta signaling. Cell. 1998;94(5):585–94. 9741623 10.1016/s0092-8674(00)81600-1

[23] Shi Y, Massague J. Mechanisms of TGF-beta signaling from cell membrane to the nucleus. Cell. 2003;113(6):685–700. 12809600 10.1016/s0092-8674(03)00432-x

[24] Macias MJ, Martin-Malpartida P, Massagué J. Structural determinants of Smad function in TGF-β signaling. Trends Biochem Sci. 2015;40(6):296–308. 25935112 10.1016/j.tibs.2015.03.012 PMC4485443

[25] Georgakopoulos-Soares I, Deng C, Agarwal V, Chan CSY, Zhao J, Inoue F, et al. Transcription factor binding site orientation and order are major drivers of gene regulatory activity. Nat Commun. 2023;14(1):2333. 37087538 10.1038/s41467-023-37960-5 PMC10122648

[26] Thanos D, Maniatis T. Virus induction of human IFN beta gene expression requires the assembly of an enhanceosome. Cell. 1995;83(7):1091–100. 8548797 10.1016/0092-8674(95)90136-1

[27] Panne D. The enhanceosome. Curr Opin Struct Biol. 2008;18(2):236–42. 18206362 10.1016/j.sbi.2007.12.002

[28] Itoh Y, Koinuma D, Omata C, Ogami T, Motizuki M, Yaguchi SI, et al. A comparative analysis of Smad-responsive motifs identifies multiple regulatory inputs for TGF-beta transcriptional activation. J Biol Chem. 2019;294(42):15466–79. 31481467 10.1074/jbc.RA119.009877 PMC6802517

[29] Morikawa M, Mitani Y, Holmborn K, Kato T, Koinuma D, Maruyama J, et al. The ALK-1/SMAD/ATOH8 axis attenuates hypoxic responses and protects against the development of pulmonary arterial hypertension. Sci Signal. 2019;12(607):eaay4430. 31719172 10.1126/scisignal.aay4430 PMC6908447

[30] Reichenbach M, Mendez PL, da Silva MC, Ugorets V, Rikeit P, Boerno S, et al. Differential impact of fluid shear stress and YAP/TAZ on BMP/TGF-beta induced osteogenic target genes. Adv Biol (Weinh). 2021;5(2):e2000051. 36073990 10.1002/adbi.202000051

[31] Ramachandran A, Vizan P, Das D, Chakravarty P, Vogt J, Rogers KW, et al. TGF-beta uses a novel mode of receptor activation to phosphorylate SMAD1/5 and induce epithelial-to-mesenchymal transition. Elife. 2018;7:7. 10.7554/eLife.31756 PMC5832415 29376829

[32] Venkatesan AM, Vyas R, Gramann AK, Dresser K, Gujja S, Bhatnagar S, et al. Ligand-activated BMP signaling inhibits cell differentiation and death to promote melanoma. J Clin Invest. 2018;128(1):294–308. 29202482 10.1172/JCI92513 PMC5749509

[33] Gunne-Braden A, Sullivan A, Gharibi B, Sheriff RSM, Maity A, Wang YF, et al. GATA3 mediates a fast, irreversible commitment to BMP4-driven differentiation in human embryonic stem cells. Cell Stem Cell. 2020;26(5):693-706 e9. 32302522 10.1016/j.stem.2020.03.005 PMC7487786

[34] Trompouki E, Bowman TV, Lawton LN, Fan ZP, Wu DC, DiBiase A, et al. Lineage regulators direct BMP and Wnt pathways to cell-specific programs during differentiation and regeneration. Cell. 2011;147(3):577–89. 22036566 10.1016/j.cell.2011.09.044 PMC3219441

[35] Ruiz L, Kaczmarska Z, Gomes T, Aragon E, Torner C, Freier R, et al. Unveiling the dimer/monomer propensities of Smad MH1-DNA complexes. Comput Struct Biotechnol J. 2021;19:632–46. 33510867 10.1016/j.csbj.2020.12.044 PMC7810915

[36] Kulkarni MM, Arnosti DN. Information display by transcriptional enhancers. Development. 2003;130(26):6569–75. 14660545 10.1242/dev.00890

[37] Song CZ, Siok TE, Gelehrter TD. Smad4/DPC4 and Smad3 mediate transforming growth factor-beta (TGF-beta) signaling through direct binding to a novel TGF-beta-responsive element in the human plasminogen activator inhibitor-1 promoter. J Biol Chem. 1998;273(45):29287–90. 9792626 10.1074/jbc.273.45.29287

[38] Johnson K, Kirkpatrick H, Comer A, Hoffmann FM, Laughon A. Interaction of Smad complexes with tripartite DNA-binding sites. J Biol Chem. 1999;274(29):20709–16. 10400705 10.1074/jbc.274.29.20709

[39] Pyrowolakis G, Hartmann B, Muller B, Basler K, Affolter M. A simple molecular complex mediates widespread BMP-induced repression during Drosophila development. Dev Cell. 2004;7(2):229–40. 15296719 10.1016/j.devcel.2004.07.008

[40] Weiss A, Charbonnier E, Ellertsdottir E, Tsirigos A, Wolf C, Schuh R, et al. A conserved activation element in BMP signaling during Drosophila development. Nat Struct Mol Biol. 2010;17(1):69–76. 20010841 10.1038/nsmb.1715

[41] Chayengia M, Veikkolainen V, Jevtic M, Pyrowolakis G. Sequence environment of BMP-dependent activating elements controls transcriptional responses to Dpp signaling in Drosophila. Development. 2019;146(11):dev176107. 31110028 10.1242/dev.176107

[42] Chai J, Wu JW, Yan N, Massague J, Pavletich NP, Shi Y. Features of a Smad3 MH1-DNA complex. Roles of water and zinc in DNA binding. J Biol Chem. 2003;278(22):20327–31. 12686552 10.1074/jbc.C300134200

[43] Ortega E, Rengachari S, Ibrahim Z, Hoghoughi N, Gaucher J, Holehouse AS, et al. Transcription factor dimerization activates the p300 acetyltransferase. Nature. 2018;562(7728):538–44. 30323286 10.1038/s41586-018-0621-1 PMC6914384

[44] Conidi A, Cazzola S, Beets K, Coddens K, Collart C, Cornelis F, et al. Few Smad proteins and many Smad-interacting proteins yield multiple functions and action modes in TGFbeta/BMP signaling in vivo. Cytokine Growth Factor Rev. 2011;22(5–6):287–300. 22119658 10.1016/j.cytogfr.2011.11.006

[45] Kasakura K, Nagata K, Miura R, Iida M, Nakaya H, Okada H, et al. Cooperative regulation of the mucosal mast cell-specific protease genes Mcpt1 and Mcpt2 by GATA and Smad transcription factors. J Immunol. 2020;204(6):1641–9. 32005755 10.4049/jimmunol.1900094 PMC8219243

[46] Nordin K, LaBonne C. Sox5 is a DNA-binding cofactor for BMP R-Smads that directs target specificity during patterning of the early ectoderm. Dev Cell. 2014;31(3):374–82. 25453832 10.1016/j.devcel.2014.10.003 PMC4255363

[47] Yan Y, Tao H, He J, Huang SY. The HDOCK server for integrated protein-protein docking. Nat Protoc. 2020;15(5):1829–52. 32269383 10.1038/s41596-020-0312-x

[48] Li H, Huang E, Zhang Y, Huang SY, Xiao Y. HDOCK update for modeling protein-RNA/DNA complex structures. Protein Sci. 2022;31(11): e4441. 36305764 10.1002/pro.4441 PMC9615301

[49] Van Der Spoel D, Lindahl E, Hess B, Groenhof G, Mark AE, Berendsen HJ. GROMACS: fast, flexible, and free. J Comput Chem. 2005;26(16):1701–18. 16211538 10.1002/jcc.20291

[50] Lindorff-Larsen K, Piana S, Palmo K, Maragakis P, Klepeis JL, Dror RO, et al. Improved side-chain torsion potentials for the Amber ff99SB protein force field. Proteins. 2010;78(8):1950–8. 20408171 10.1002/prot.22711 PMC2970904

[51] Cornell WD, Cieplak P, Bayly CI, Gould IR, Merz KM, Ferguson DM, et al. A second generation force field for the simulation of proteins, nucleic acids, and organic molecules. J. Am. Chem. Soc. 1995, 117, 5179−5197. Journal of the American Chemical Society. 1996;118(9):2309-.

[52] Zou Z, Ohta T, Miura F, Oki S. ChIP-Atlas 2021 update: a data-mining suite for exploring epigenomic landscapes by fully integrating ChIP-seq, ATAC-seq and Bisulfite-seq data. Nucleic Acids Res. 2022;50(W1):W175–82. 35325188 10.1093/nar/gkac199 PMC9252733

[53] Heinz S, Benner C, Spann N, Bertolino E, Lin YC, Laslo P, et al. Simple combinations of lineage-determining transcription factors prime cis-regulatory elements required for macrophage and B cell identities. Mol Cell. 2010;38(4):576–89. 20513432 10.1016/j.molcel.2010.05.004 PMC2898526

