# Supplementary material for: Rare but specific: 5-bp composite motifs define SMAD binding in BMP signaling
Source: BMC Biol. 2025 Mar 13;23:79. doi: 10.1186/s12915-025-02183-1 (PMC11907993; doi:10.1186/s12915-025-02183-1)
Supplement: Supplementary file 1 — Additional file 1: Supplementary Figs. S1–S7. Fig. S1 SMAD-MH1 purity control and experimental set-up. Fig. S2 BRE2-Luc and CAGA12-Luc define experimental conditions for selective comparison of BMP6 and TGFβ1. Fig. S3 BMP6 dose–response curves of SMAD composite motif reporters. Fig. S4 Composite motif spacers below 5 bp do not inhibit SMAD1-MH1 double binding. Fig. S5 BMP-responsive SMAD homocomposite motif reporters are unresponsive to TGFβ1. Fig. S6 BMP-responsive SMAD heterocomposite motif reporters are unresponsive to TGFβ1. Fig. S7 BMP specificity of SMAD composite motif reporters is maintained in U2OS cells. [file 12915_2025_2183_MOESM1_ESM.pdf]

Rare but specific:
5-bp Composite Motifs Define SMAD Binding in BMP Signaling

Jerome Jatzlau[1,2], Sophie-Nhi Do[1], Rebeca A. Mees[2], Paul-Lennard Mendez[1], Rameez Jabeer Khan[2], Lukas Maas[1], Lidia Ruiz[2], Pau Martin-Malpartida[2], Maria J. Macias[2,3,*], Petra Knaus[1,*]

Supplementary Figures

Fig. S1. SMAD-MH1 purity control and experimental set-up

Fig. S2. BRE_2-Luc and $CAGA_{12}$-Luc define experimental conditions for selective comparison of BMP6 and TGFβ1

Fig. S3. BMP6 dose-response curves of SMAD composite motif reporters

Fig. S4. Composite motif spacers below 5 bp do not inhibit SMAD1-MH1 double binding

Fig. S5. BMP-responsive SMAD homocomposite motif reporters are unresponsive to TGFβ1

Fig. S6. BMP-responsive SMAD heterocomposite motif reporters are unresponsive to TGFβ1

Fig. S7. BMP-specificity of SMAD composite motif reporters is maintained in U2OS cells

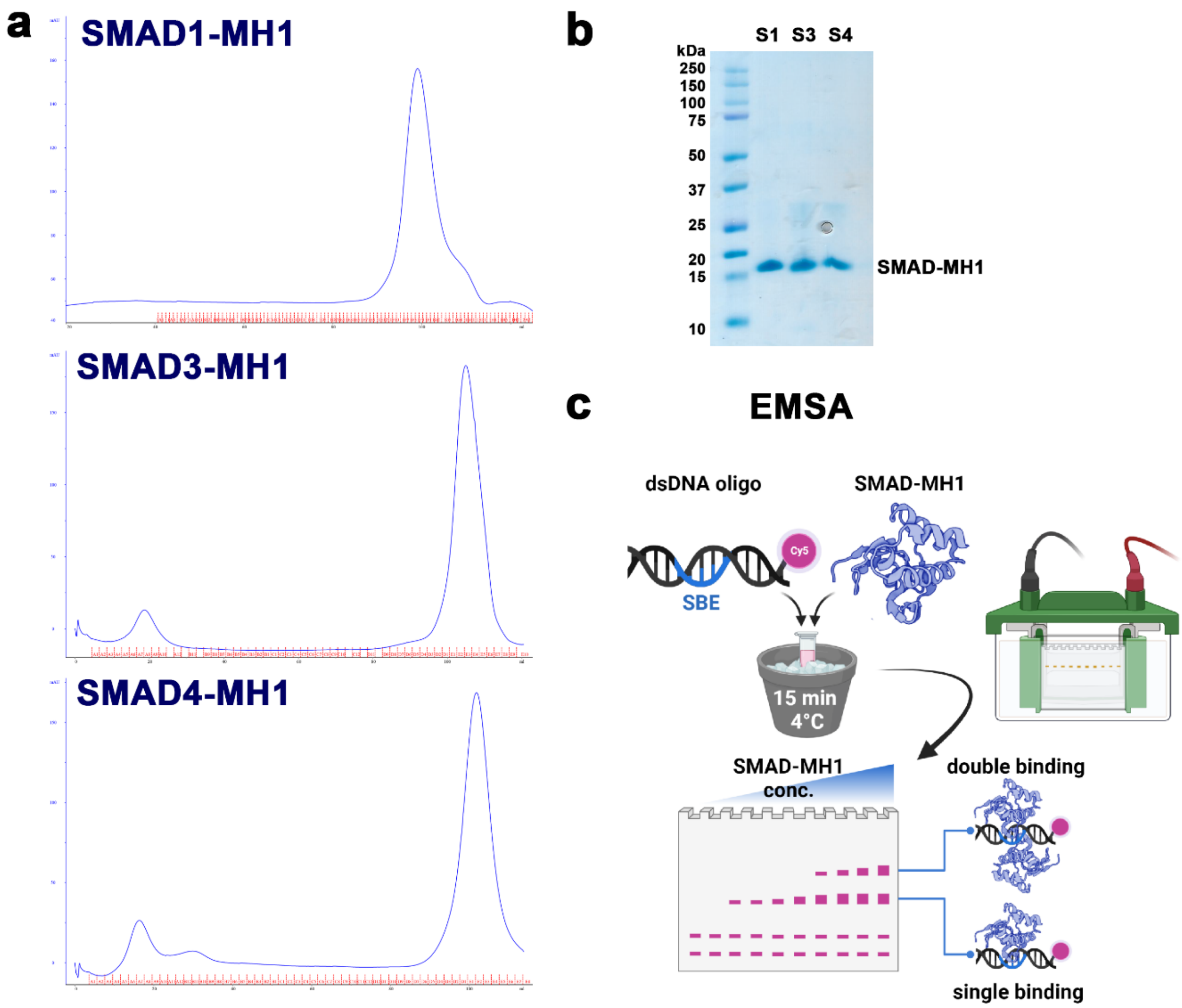

Fig. S1. SMAD-MH1 purity control and experimental set-up: ***a***, *SEC traces of purified SMAD-MH1 domains.* ***b,*** *Coomassie staining of purified SMAD-MH1 domains.* ***c,*** *Increasing concentration of SMAD1/3/4-MH1 domains were incubated with Cy5-dsDNA oligos containing known SMAD-binding element (SBE)s and analyzed for binding using Electro Mobility Shift Assay. Single or double binding of SMAD-MH1 is detected by weight shift of Cy5-dsDNA signal.*

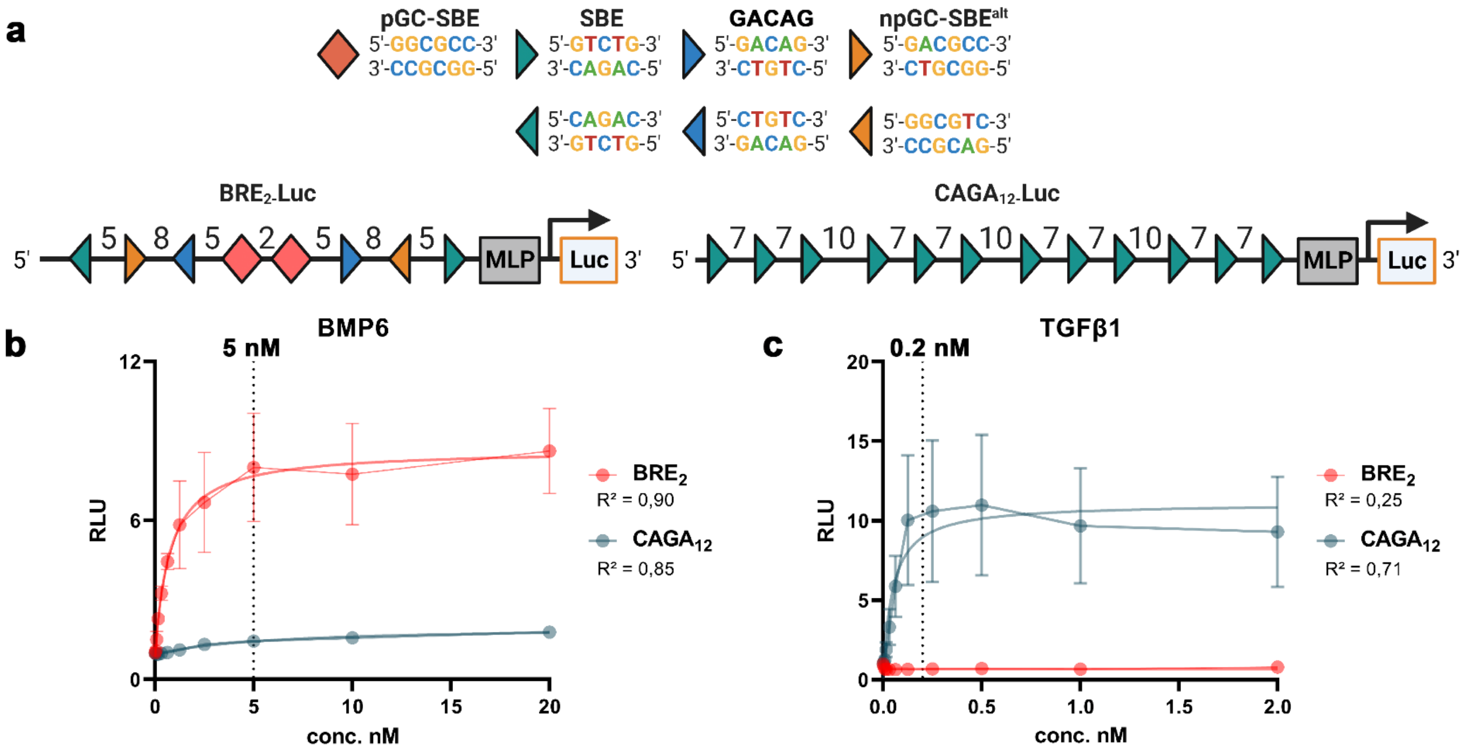

Fig. S2. BRE₂-Luc and CAGA₁₂-Luc define experimental conditions for selective comparison of BMP6 and TGFβ1: ***a***, *SMAD motifs in the two luciferase reporters are indicated.* ***b,*** *BRE$_2$-Luc or CAGA$_{12}$-Luc was transfected together with TK-renilla luciferase into HEK293t cells, cells were then starved and stimulated with BMP6 (0.03 - 20 nM) or TGFβ1 (0.003 - 2 nM) for 24 h before analysis using a microplate reader.* ***b-c,*** *Data are shown as mean fold induction in relative luciferase units (RLU) ±SD (n=3 independent experiments). 5 nM BMP6 and 0.2 nM TGFβ1 were selected for all consecutive experiments as they gave a high response close to saturation of the signal.*

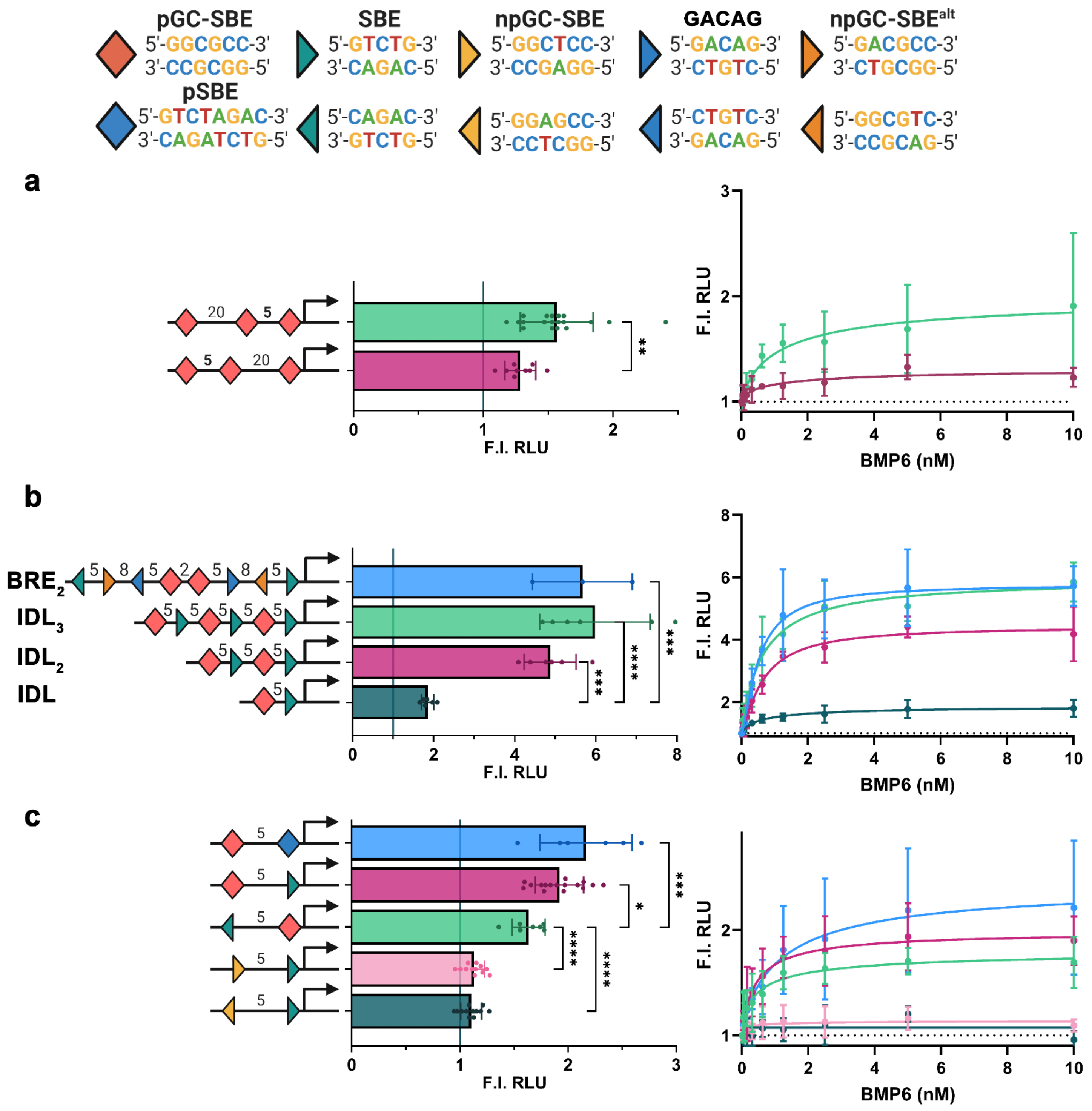

Fig. S3. BMP6 dose-response curves of SMAD composite motif reporters : ***a-c,*** *HEK293t cells were co-transfected with SBE-firefly-Luc constructs and TK-renilla luciferase, starved and stimulated with BMP6 for 24 hrs before analysis using a microplate reader. Bar charts represent response to 5 nM BMP6 (left) and dose curves represent the response to 0.04 nM to 10 nM BMP6 (right).* ***a****, Dual luciferase reporter assay displaying decreased BMP6-responsiveness, if 5bp-spaced pGC-SBE*

homocomposite motif is 20+6 bp further away from the MLP. ***b****, Dual luciferase reporter assay displaying elevated BMP6-responsiveness towards multiplied (2-3x) pGC-SBE/SBE heterocomposite motifs.* ***c****, Dose response comparison of heterocomposite motifs from Fig.3.* ***a-c****, Data are shown as mean fold induction to unstimulated cells (grey line) in relative luciferase units (RLU) ±SD (5nM BMP6 n=3–19; dose curves n=3 independent experiments). Statistical significance was calculated between samples using one-way ANOVA and Tukey's post-hoc test.*

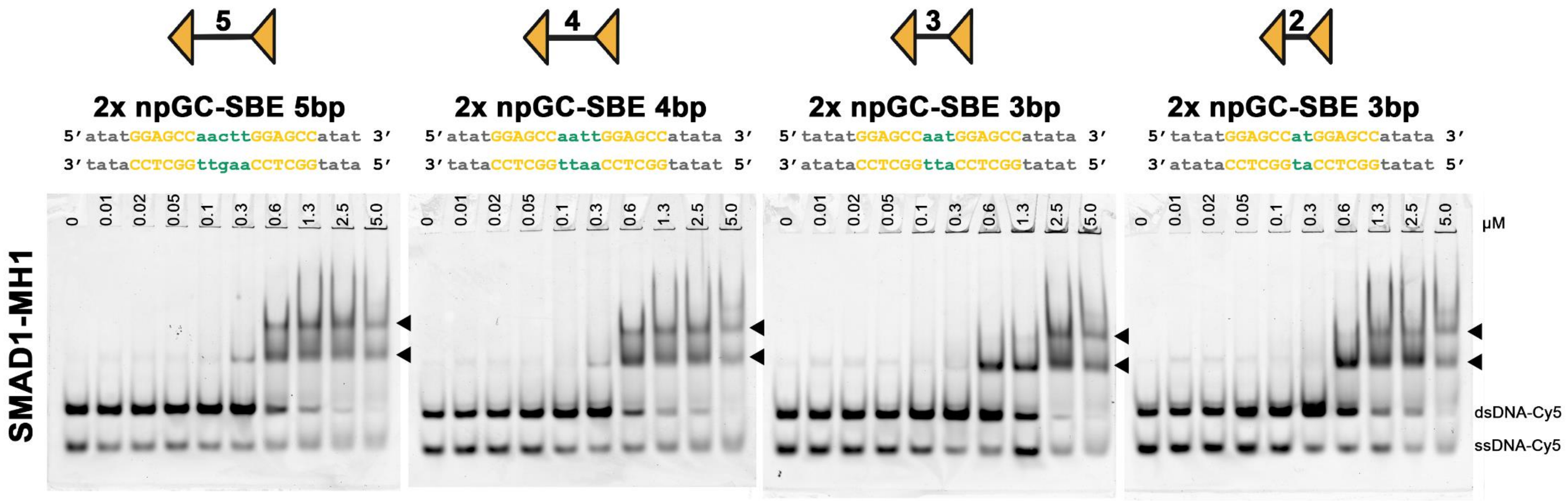

Fig. S4. Composite motif spacers below 5 bp do not inhibit SMAD1-MH1 double binding: *EMSA experiments were performed testing the binding of human SMAD1-MH1 domains to differently spaced (2-5 bp) npGC-SBE homocomposite motifs. Protein concentrations (μM) are shown on top of the EMSA. Abbreviations for the DNA oligonucleotides and dsDNA sequence are shown above. Single and double SMAD-MH1 binding to dsDNA is indicated with black triangles.*

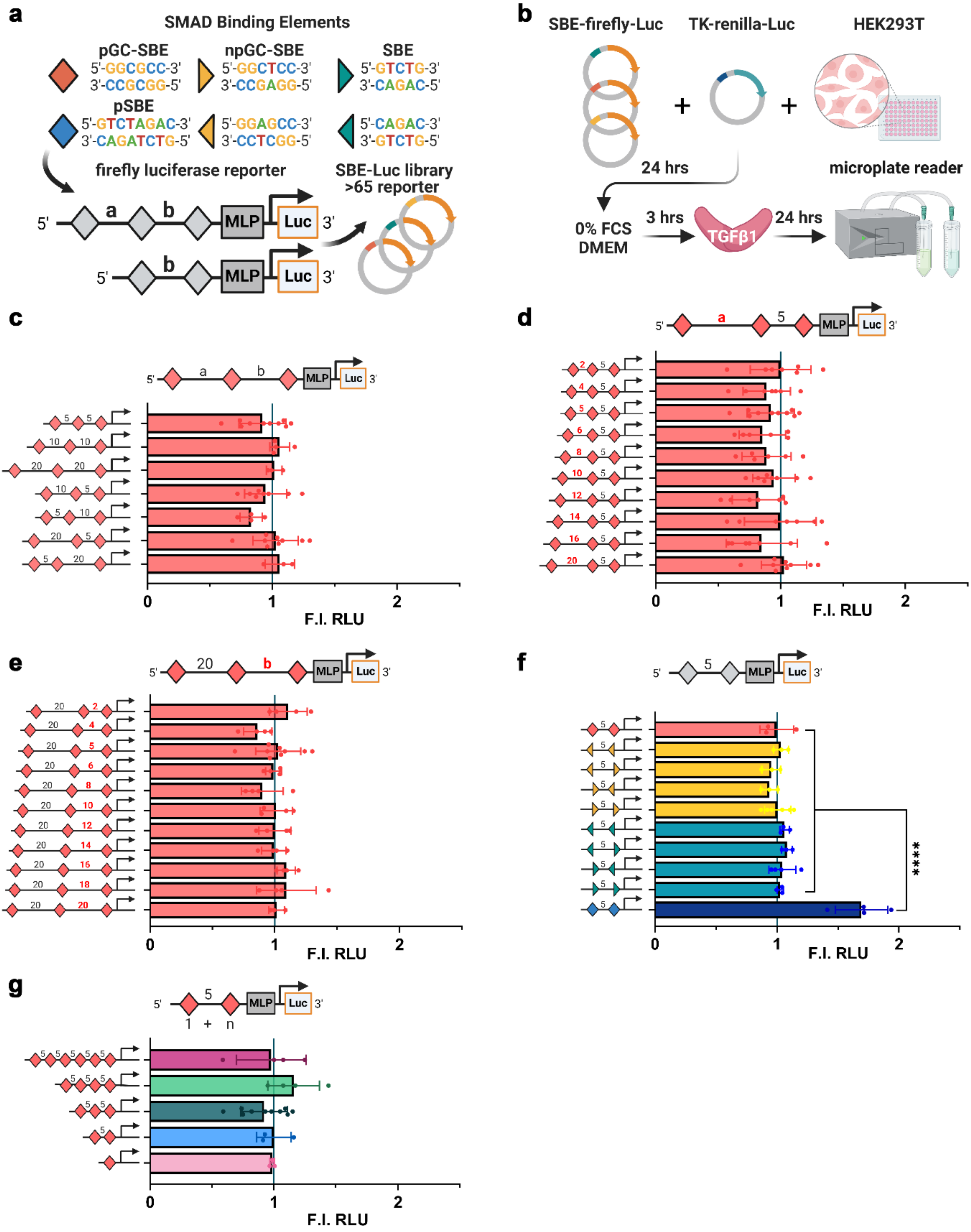
a
SMAD Binding Elements
pGC-SBE
5'-GGCGCC-3'
3'-CCGCGG-5'
npGC-SBE
5'-GGCTCC-3'
3'-CCGAGG-5'
SBE
5'-GTCTG-3'
3'-CAGAC-5'
pSBE
5'-GTCTAGAC-3'
3'-CAGATCTG-5'
5'-GGAGCC-3'
3'-CCTCGG-5'
5'-CAGAC-3'
3'-GTCTG-5'
firefly luciferase reporter
SBE-Luc library
>65 reporter
MLP
Luc
b
SBE-firefly-Luc
TK-renilla-Luc
HEK293T
24 hrs
microplate reader
0% FCS
DMEM
3 hrs
TGFβ1
24 hrs
c
d
e
f
g
F.I. RLU
****

Fig. S5. BMP-responsive SMAD homocomposite motif reporters are unresponsive to TGFβ1: ***a***, *A library of synthetic firefly luciferase constructs was cloned with 1 to 6 SMAD motifs positioned 10 bp before a minimal promotor (MLP) with varying spacer length for pGC-SBEs and varying orientation for npGC-SBE and SBE motifs.* ***b***, *HEK293t cells were co-transfected with SBE-firefly-Luc constructs and TK-renilla luciferase, starved and stimulated with TGFβ1 (0.2 nM) for 24 hrs before analysis using a microplate reader.* ***c-g***, *Dual luciferase reporter assay displaying no TGFβ1-responsiveness towards constructs with differently spaced SMAD homocomposite motifs, except to two 5-bp spaced pSBE motifs.* ***c-g***, *Data are shown as mean fold induction to unstimulated cells (grey line) in relative luciferase units (RLU) ±SD (n=3–10 independent experiments). Statistical significance was calculated between samples using one-way ANOVA and Tukey's post-hoc test.*

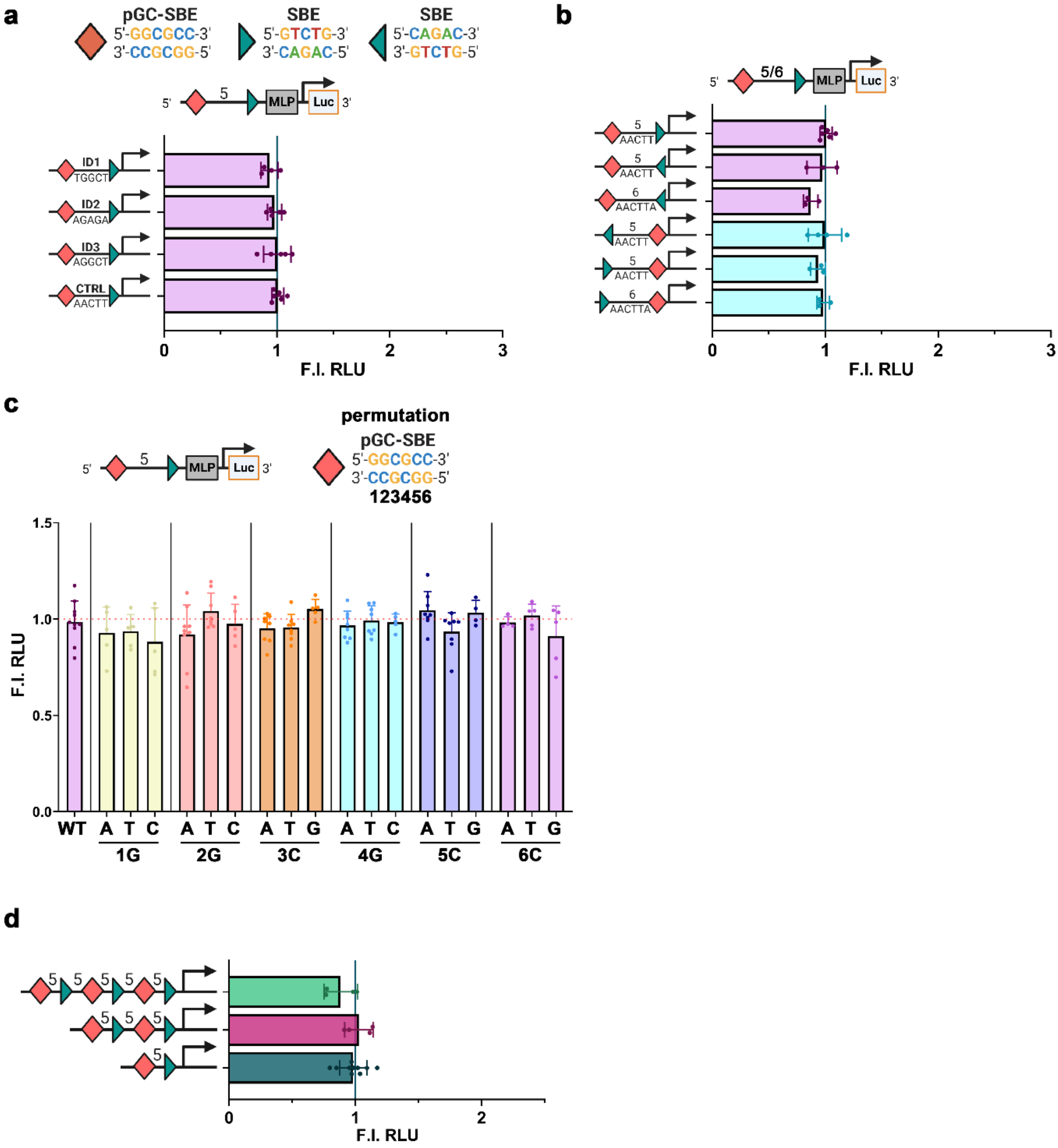

Fig. S6. BMP-responsive SMAD heterocomposite motif reporters are unresponsive to TGFβ1: ***a-c****, Dual luciferase reporter assay displaying no TGFβ1-responsiveness (0.2 nM TGFβ1, 24 hrs) towards constructs with differently spaced (****a****), oriented (****b****), or permutated (****c****) or multiplied (****d****) SMAD heterocomposite motifs. Data are shown as mean fold induction to unstimulated cells (line) in relative luciferase units (RLU) ±SD (n=3–10 independent experiments). Statistical significance was calculated between samples using one-way ANOVA and Tukey's post-hoc test (****a-b,d****) and between samples (****c****) or relative to WT ctrl (****c****) using one-way ANOVA and Šídák's or Dunnett's multiple comparisons test.*

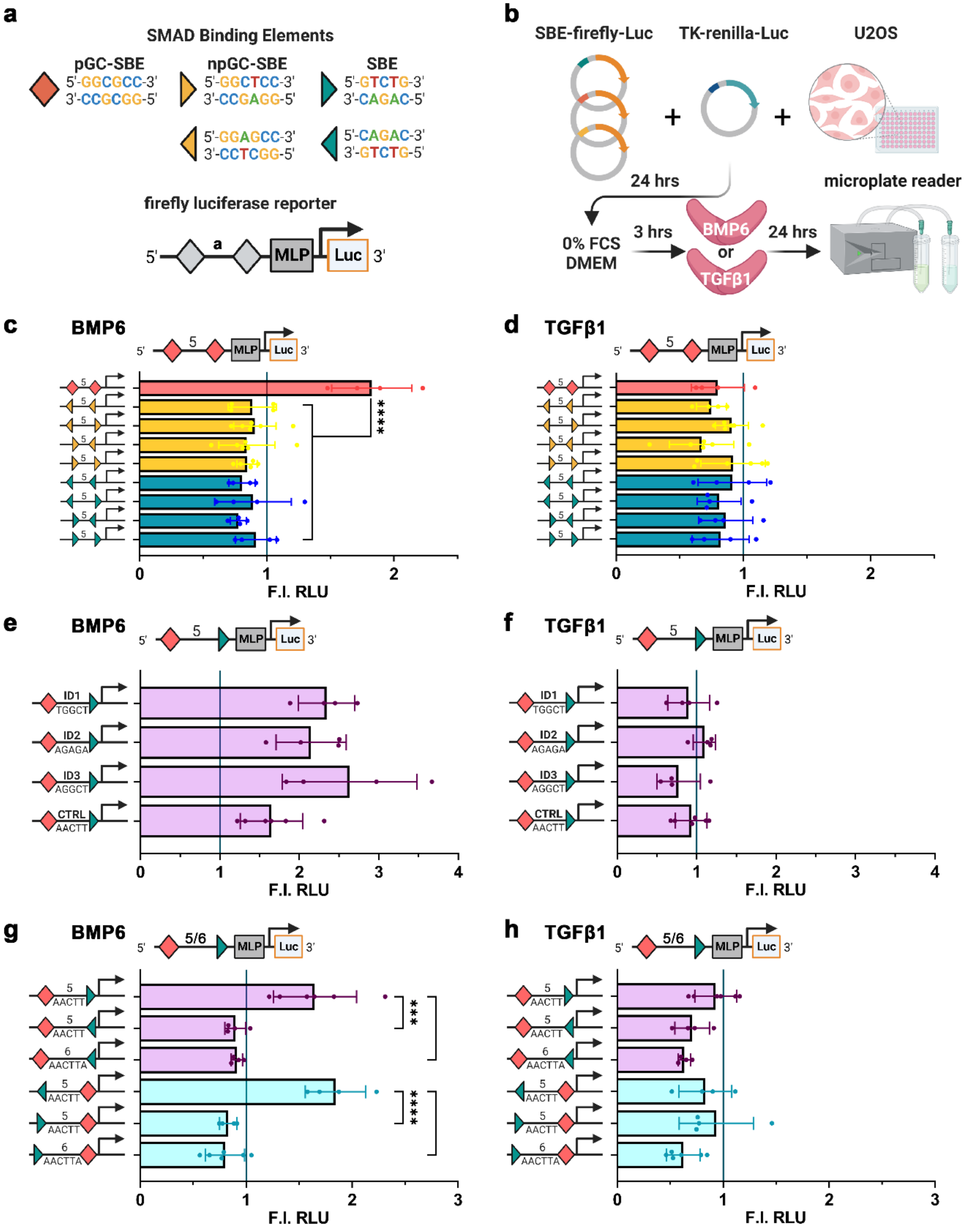
a
SMAD Binding Elements
pGC-SBE
5'-GGCGCC-3'
3'-CCGCGG-5'
npGC-SBE
5'-GGCTCC-3'
3'-CCGAGG-5'
SBE
5'-GTCTG-3'
3'-CAGAC-5'
5'-GGAGCC-3'
3'-CCTCGG-5'
5'-CAGAC-3'
3'-GTCTG-5'
firefly luciferase reporter
5' a MLP Luc 3'
b
SBE-firefly-Luc
TK-renilla-Luc
U2OS
24 hrs
microplate reader
0% FCS DMEM
3 hrs
BMP6 or TGFβ1
24 hrs
c
BMP6
d
TGFβ1
F.I. RLU
e
BMP6
f
TGFβ1
ID1 TGGCT
ID2 AGAGA
ID3 AGGCT
CTRL AACTT
g
BMP6
h
TGFβ1
5/6
AACTT
AACTTA

Fig. S7. BMP-specificity of SMAD composite motif reporters is maintained in U2OS cells: ***a****, A library of synthetic firefly luciferase construct was cloned with 2 SMAD motifs positioned before a minimal promotor (MLP) with varying spacer length and orientation of SMAD motifs.* ***b****, U2OS cells were co-transfected with SBE-firefly-Luc constructs and TK-renilla luciferase, starved, and stimulated with BMP6 (5 nM) or TGFβ1 (0.2 nM) for 24 hrs before analysis using a microplate reader.* ***c-h****, Dual luciferase reporter assay displaying BMP6-responsiveness towards 5-bp spaced SMAD homo- and heterocomposite motif constructs. Data are shown as mean fold induction to unstimulated cells in relative luciferase units (RLU) ±SD (n=4–6 independent experiments). Statistical significance was calculated between samples using one-way ANOVA and Tukey's post-hoc test.*
