# Supplementary material for: Rare but specific: 5-bp composite motifs define SMAD binding in BMP signaling
Source: BMC Biol. 2025 Mar 13;23:79. doi: 10.1186/s12915-025-02183-1 (PMC11907993; doi:10.1186/s12915-025-02183-1)
Supplement: Supplementary file 3 — Additional file 3: Table S2 DNA oligo sequences. [file 12915_2025_2183_MOESM3_ESM.pdf]

Tabel 2 Oligo sequences for GC-SBE luciferase reporter library

annealed oligos carry XhoI and KpnI overhangs

pGC-SBE (GGCGCC), npGC-SBE (GGAGCC/GGCTCC), SBE(GTCTG/CAGAC), pSBE(GTCTAGAC)

permutated pGC-SBE (GGCGCC) 1-6 position exchanged against A/T/G

Oligo Nr.	for/ rev	Sequence (5' → 3')
1	for	CGGCGCCCACTGGAACTGTTGACTGCGGGCGCCAACTTGGCGCCC
1	rev	TCGAGGGCGCCAAGTTGGCGCCCGCAGTCAACAGTTCCAGTGGGCGCCGGTAC
2	for	CGGCGCCCACTGGAACTGTTGACTGCGGGCGCCGTGGCGCCC
2	rev	TCGAGGGCGCCACGGCGCCCGCAGTCAACAGTTCCAGTGGGCGCCGGTAC
3	for	CGGCGCCCACTGGAACTGTTGACTGCGGGCGCCGCATGGCGCCC
3	rev	TCGAGGGCGCCATGCGGCGCCCGCAGTCAACAGTTCCAGTGGGCGCCGGTAC
4	for	CGGCGCCCACTGGAACTGTTGACTGCGGGCGCCGCAGATGGCGCCC
4	rev	TCGAGGGCGCCATCTGCGGCGCCCGCAGTCAACAGTTCCAGTGGGCGCCGGTAC
5	for	CGGCGCCCACTGGAACTGTTGACTGCGGGCGCCGCAGTGATGGCGCCC
5	rev	TCGAGGGCGCCATCACTGCGGCGCCCGCAGTCAACAGTTCCAGTGGGCGCCGGTAC
6	for	CGGCGCCCACTGGAACTGTTGACTGCGGGCGCCGCAGTATCTGATGGCGCCC
6	rev	TCGAGGGCGCCATCAGATACTGCGGCGCCCGCAGTCAACAGTTCCAGTGGGCGCCGGTAC
7	for	CGGCGCCCACTGGAACTGTTGACTGCGGGCGCCGCAGTCAATCTGATGGCGCCC
7	rev	TCGAGGGCGCCATCAGATTGACTGCGGCGCCCGCAGTCAACAGTTCCAGTGGGCGCCGGTAC
8	for	CGGCGCCCACTGGAACTGTTGACTGCGGGCGCCGCAGTCAACTTCTGATGGCGCCC
8	rev	TCGAGGGCGCCATCAGAAGTTGACTGCGGCGCCCGCAGTCAACAGTTCCAGTGGGCGCCGGTAC
9	for	CGGCGCCCACTGGAACTGTTGACTGCGGGCGCCGCAGTCGCAACTTCTGATGGCGCCC
9	rev	TCGAGGGCGCCATCAGAAGTTGCGACTGCGGCGCCCGCAGTCAACAGTTCCAGTGGGCGCCGGTAC
10	for	CGGCGCCCACCGGGCGCCAACTTGGCGCCC
10	rev	TCGAGGGCGCCAAGTTGGCGCCCGGTGGGCGCCGGTAC
11	for	CGGCGCCCACTGGTGCGGGCGCCAACTTGGCGCCC
11	rev	TCGAGGGCGCCAAGTTGGCGCCCGCACCAGTGGGCGCCGGTAC
12	for	CGGCGCCCACCGGGCGCCGCAGTCGCGCAACTTCTGATGGCGCCC
12	rev	TCGAGGGCGCCATCAGAAGTTGCGCGACTGCGGCGCCCGGTGGGCGCCGGTAC
13	for	CAATACTCACTGGAACTGTTGACTGCGGGCGCCAACTTGGCGCCC
13	rev	TCGAGGGCGCCAAGTTGGCGCCCGCAGTCAACAGTTCCAGTGAGTATTGGTAC
14	for	CGGCGCCCACCGGGCGCCCACCGAACTTGGCGCCC
14	rev	TCGAGGGCGCCAAGTTCGGTGGGCGCCCGGTGGGCGCCGGTAC
15	for	CAATACTCACCGGGCGCCAACTTGGCGCCC
15	rev	TCGAGGGCGCCAAGTTGGCGCCCGGTGAGTATTGGTAC
16	for	CGGCGCCCACCGAATACTAACTTGGCGCCC
16	rev	TCGAGGGCGCCAAGTTAGTATTCGGTGGGCGCCGGTAC
17	for	CGGCGCCCACCGGGCGCCAACTTAATACTC
17	rev	TCGAGAGTATTAAGTTGGCGCCCGGTGGGCGCCGGTAC
18	for	CGGCGCCCAGGCGCCGCGATGGCGCCC
18	rev	TCGAGGGCGCCATCGCGGCGCCTGGGCGCCGGTAC
19	for	CGGCGCCCACGGCGCCGCGATGGCGCCC
19	rev	TCGAGGGCGCCATCGCGGCGCCCGTGGGCGCCGGTAC
20	for	CGGCGCCCACACGGGCGCCGCGATGGCGCCC
20	rev	TCGAGGGCGCCATCGCGGCGCCCGTGTGGGCGCCGGTAC
21	for	CGGCGCCCACTTACGGGCGCCGCGATGGCGCCC
21	rev	TCGAGGGCGCCATCGCGGCGCCCGTAAGTGGGCGCCGGTAC
22	for	CGGCGCCCACTGGTTGACGGGCGCCGCGATGGCGCCC
22	rev	TCGAGGGCGCCATCGCGGCGCCCGTCAACCAGTGGGCGCCGGTAC
23	for	CGGCGCCCACTGGTTGATGCGGGCGCCGCGATGGCGCCC
23	rev	TCGAGGGCGCCATCGCGGCGCCCGCATCAACCAGTGGGCGCCGGTAC
24	for	CGGCGCCCACTGGACTTGATGCGGGCGCCGCGATGGCGCCC
24	rev	TCGAGGGCGCCATCGCGGCGCCCGCATCAAGTCCAGTGGGCGCCGGTAC

**25**	for	CGGCGCCCACTGGACTGTTGATGCGGGCGCCGCGATGGCGCCC
**25**	rev	TCGAGGGCGCCATCGCGGCGCCCGCATCAACAGTCCAGTGGGCGCCGGTAC
**26**	for	CGGCGCCTGGCTGTCTGC
**26**	rev	TCGAGCAGACAGCCAGGCGCCGGTAC
**27**	for	CGGCGCCAGAGAGTCTGC
**27**	rev	TCGAGCAGACTCTCTGGCGCCGGTAC
**28**	for	CGGCGCCAGGCTGTCTGC
**28**	rev	TCGAGCAGACAGCCTGGCGCCGGTAC
**29**	for	CGGCGCCAACTTGTCTGC
**29**	rev	TCGAGCAGACAAGTTGGCGCCGGTAC
**30**	for	CGTCTGAACTTGGCGCCC
**30**	rev	TCGAGGGCGCCAAGTTCAGACGGTAC
**31**	for	CGTCTGTAACTTGGCGCCC
**31**	rev	TCGAGGGCGCCAAGTTACAGACGGTAC
**32**	for	CCAGACAACTTGGCGCCC
**32**	rev	TCGAGGGCGCCAAGTTGTCTGGGTAC
**33**	for	CGGCGCCAACTTCAGACC
**33**	rev	TCGAGGTCTGAAGTTGGCGCCGGTAC
**34**	for	CGGCGCCAACTTACAGACC
**34**	rev	TCGAGGTCTGTAAGTTGGCGCCGGTAC
**35**	for	CGGAGCCAACTTGGAGCCC
**35**	rev	TCGAGGGCTCCAAGTTGGCTCCGGTAC
**36**	for	CGGAGCCAACTTGGCTCCC
**36**	rev	TCGAGGGAGCCAAGTTGGCTCCGGTAC
**37**	for	CGGCTCCAACTTGGAGCCC
**37**	rev	TCGAGGGCTCCAAGTTGGAGCCGGTAC
**38**	for	CGGCTCCAACTTGGCTCCC
**38**	rev	TCGAGGGAGCCAAGTTGGAGCCGGTAC
**39**	for	CGGCGCCAACTTGGCGCCC
**39**	rev	TCGAGGGCGCCAAGTTGGCGCCGGTAC
**40**	for	CGGCTCCAACTTGTCTGC
**40**	rev	TCGAGCAGACAAGTTGGAGCCGGTAC
**41**	for	CGGAGCCAACTTGTCTGC
**41**	rev	TCGAGCAGACAAGTTGGCTCCGGTAC
**42**	for	CGTCTGAACTTGTCTGC
**42**	rev	TCGAGCAGACAAGTTCAGACGGTAC
**43**	for	CGTCTGAACTTCAGACC
**43**	rev	TCGAGGTCTGAAGTTCAGACGGTAC
**44**	for	CCAGACAACTTGTCTGC
**44**	rev	TCGAGCAGACAAGTTGTCTGGGTAC
**45**	for	CCAGACAACTTCAGACC
**45**	rev	TCGAGGTCTGAAGTTGTCTGGGTAC
**46**	for	CGACGCCAACTTGTCTGC
**46**	rev	TCGAGCAGACAAGTTGGCGTCGGTAC
**47**	for	CGTCGCCAACTTGTCTGC
**47**	rev	TCGAGCAGACAAGTTGGCGACGGTAC
**48**	for	CGCCGCCAACTTGTCTGC
**48**	rev	TCGAGCAGACAAGTTGGCGGCGGTAC
**49**	for	CGGCACCAACTTGTCTGC
**49**	rev	TCGAGCAGACAAGTTGGTGCCGGTAC
**50**	for	CGGCCCCAACTTGTCTGC
**50**	rev	TCGAGCAGACAAGTTGGGGCCGGTAC
**51**	for	CGGCGGCAACTTGTCTGC
**51**	rev	TCGAGCAGACAAGTTGCCGCCGGTAC
**52**	for	CGGCGACAACTTGTCTGC
**52**	rev	TCGAGCAGACAAGTTGTCGCCGGTAC
**53**	for	CGGCGTCAACTTGTCTGC

**53**	rev	TCGAG**CAGAC**AAGTT**GACGCC**GGTAC
**54**	for	C**GGTGCC**AACTT**GTCTG**C
**54**	rev	TCGAG**CAGAC**AAGTT**GGCACC**GGTAC
**55**	for	C**GGGGCC**AACTT**GTCTG**C
**55**	rev	TCGAG**CAGAC**AAGTT**GGCCCC**GGTAC
**56**	for	C**AGCGCC**AACTT**GTCTG**C
**56**	rev	TCGAG**CAGAC**AAGTT**GGCGCT**GGTAC
**57**	for	C**TGCGCC**AACTT**GTCTG**C
**57**	rev	TCGAG**CAGAC**AAGTT**GGCGCA**GGTAC
**58**	for	C**CGCGCC**AACTT**GTCTG**C
**58**	rev	TCGAG**CAGAC**AAGTT**GGCGCG**GGTAC
**59**	for	C**GGCGCA**AACTT**GTCTG**C
**59**	rev	TCGAG**CAGAC**AAGTT**TGCGCC**GGTAC
**60**	for	C**GGCGCT**AACTT**GTCTG**C
**60**	rev	TCGAG**CAGAC**AAGTT**AGCGCC**GGTAC
**61**	for	C**GGCGCG**AACTT**GTCTG**C
**61**	rev	TCGAG**CAGAC**AAGTT**CGCGCC**GGTAC
**62**	for	C**GGCGCC**CACCG**GGCGCC**AACTT**GGCGCC**TTATT**GGCGCC**CACCG**GGCGCC**AACTT**GGCGCC**C
**62**	rev	TCGAG**GGCGCC**AAGTT**GGCGCC**CGGTG**GGCGCC**AATAA**GGCGCC**AAGTT**GGCGCC**CGGTG**GGCGCC** GGTAC
**63**	for	C**GGCGCC**TTATT**GGCGCC**CACCG**GGCGCC**AACTT**GGCGCC**C
**63**	rev	TCGAG**GGCGCC**AAGTT**GGCGCC**CGGTG**GGCGCC**AATAA**GGCGCC**GGTAC
**64**	for	C**GGCGCC**CACCG**GTCTG**AACTT**GGCGCC**TTATT**GTCTG**CACCG**GGCGCC**AACTT**GTCTG**C
**64**	rev	TCGAG**CAGAC**AAGTT**GGCGCC**CGGTG**CAGAC**AATAA**GGCGCC**AAGTT**CAGAC**CGGTG**GGCGCC**GGTA C
**65**	for	C**GGCGCC**TTATT**GTCTG**CACCG**GGCGCC**AACTT**GTCTG**C
**65**	rev	TCGAG**CAGAC**AAGTT**GGCGCC**CGGTG**CAGAC**AATAA**GGCGCC**GGTAC
**66**	for	C**GGCGCC**AACTT**GTCTAGAC**
**66**	rev	TCGA**GTCTAGAC**AAGTT**GGCGCC**GGTAC
**67**	for	C**GTCTAGAC**AACTT**GTCTAGAC**
**67**	rev	TCGA**GTCTAGAC**AAGTT**GTCTAGAC**GGTAC
**68**	for	CTTGTTTACAACTT**GGCGCC**C
**68**	rev	TCGAG**GGCGCC**AAGTTGTAAACAAGGTAC
